# Structural basis for cellobiose dehydrogenase action during oxidative cellulose degradation

**DOI:** 10.1038/ncomms8542

**Published:** 2015-07-07

**Authors:** Tien-Chye Tan, Daniel Kracher, Rosaria Gandini, Christoph Sygmund, Roman Kittl, Dietmar Haltrich, B. Martin Hällberg, Roland Ludwig, Christina Divne

**Affiliations:** 1 School of Biotechnology, KTH Royal Institute of Technology, AlbaNova University Center, Roslagstullsbacken 21, Stockholm S-10691, Sweden; 2 Department of Medical Biochemistry and Biophysics, Karolinska Institutet, Scheelelaboratoriet, Scheeles väg 2, Stockholm S-17177, Sweden; 3 Food Biotechnology Laboratory, Department of Food Science and Technology, Vienna Institute of Biotechnology (VIBT), BOKU—University of Natural Resources and Life Sciences, Muthgasse 18, Vienna A-1190, Austria; 4 Department of Cell and Molecular Biology, Karolinska Institutet, Stockholm S-17177, Sweden; 5 European Molecular Biology Laboratory, Hamburg Unit, Hamburg 22603, Germany; and Centre for Structural Systems Biology (CSSB), DESY-Campus, Hamburg 22603, Germany

## Abstract

A new paradigm for cellulose depolymerization by fungi focuses on an oxidative mechanism involving cellobiose dehydrogenases (CDH) and copper-dependent lytic polysaccharide monooxygenases (LPMO); however, mechanistic studies have been hampered by the lack of structural information regarding CDH. CDH contains a haem-binding cytochrome (CYT) connected via a flexible linker to a flavin-dependent dehydrogenase (DH). Electrons are generated from cellobiose oxidation catalysed by DH and shuttled via CYT to LPMO. Here we present structural analyses that provide a comprehensive picture of CDH conformers, which govern the electron transfer between redox centres. Using structure-based site-directed mutagenesis, rapid kinetics analysis and molecular docking, we demonstrate that flavin-to-haem interdomain electron transfer (IET) is enabled by a haem propionate group and that rapid IET requires a closed CDH state in which the propionate is tightly enfolded by DH. Following haem reduction, CYT reduces LPMO to initiate oxygen activation at the copper centre and subsequent cellulose depolymerization.

## Abstract (web summary)

Cellobiose dehydrogenases (CDHs) cooperate with lytic polysaccharide monooxygenases (LPMOs) to catalyse cellulose degradation. Here Tan *et al*. define the electron transfer pathway in CDH, providing a structural analysis of CDH conformers and of the interaction between CDH and LPMO during cellulose depolymerisation.

## Graphical abstract

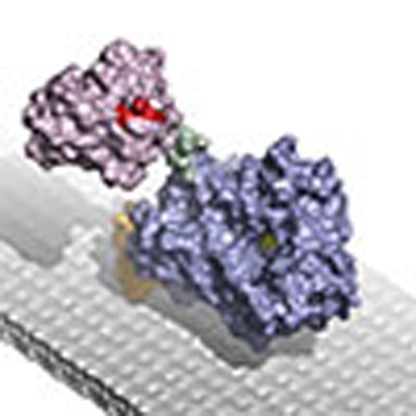

The need for renewable energy is increasing rapidly, and biofuel derived from plant matter is an attractive alternative to fossil-based fuels. However, the bioconversion of the major component of plant matter, cellulose, to low-molecular-weight saccharides is problematic and costly^1^,^2^. Despite decades of research on the molecular mechanisms of microbial cellulose depolymerization, a comprehensive picture of this elaborate biodegradation machinery has remained elusive. In nature, rot fungi and bacteria are primary factors in the recycling of lignocellulose-based biomass, and the efficient saccharification of cellulose has historically been assigned to a cascade of hydrolytic enzymes. An oxidative system was recently discovered in which extracellular flavocytochromes, that is, cellobiose dehydrogenases (CDHs)^3^,^4^,^5^,^6^,^7^, cooperate with copper-dependent lytic polysaccharide monooxygenases (LPMOs)^8^,^9^,^10^,^11^,^12^,^13^ to catalyse redox-mediated glycosidic bond cleavage in crystalline cellulose, hemicelluloses and starch. The CDH-LPMO system enhances the degradation efficiency of crystalline regions in cellulose by a previously unknown mechanism^14^,^15^,^16^,^17^,^18^,^19^.

CDHs are large flavocytochromes containing a haem *b*-binding cytochrome domain (CYT) connected by a long, flexible linker to a flavin adenine dinucleotide (FAD)-binding dehydrogenase domain (DH)^20^. Class-I CDHs are produced by basidiomycetes and lack additional domains, whereas class-II CDHs occur in ascomycetes either with or without a type-1 carbohydrate-binding module (CBM), corresponding to classes IIA and IIB, respectively^20^,^21^. Despite the absence of a CBM, class-I CDHs bind strongly to the cellulose surface by an unknown mechanism^22^. The DH domain oxidizes cellobiose at the C1 position to cellobiono-1,5-lactone with reduction of FAD. The ensuing step involves inter-domain electron transfer (IET) from the reduced FAD to CYT haem *b*, presumably by single electron-transfer (ET) events, followed by ET from CYT to external electron acceptors^20^ such as LPMOs. LPMOs directly hydroxylate the crystalline polysaccharide substrate at C1 or C4 to produce the aldonic acid or the 4-keto sugar, respectively^10^,^17^. The precise mechanism of the monooxygenation reaction is unknown but likely involves C-H activation in which hydrogen abstraction and the formation of a radical oxygen species enable substrate hydroxylation, either by a superoxo mechanism^15^,^17^,^23^ or by an oxyl mechanism^24^.

As the linker between CYT and DH is long and flexible, attempts to crystallize full-length CDHs have been unsuccessful. For the CDH from the basidiomycete *Phanerochaete chrysosporium* (*Pc*CDH), crystal structures of the proteolytically generated CYT and DH fragments were determined separately^25^,^26^, but these studies did not provide experimental information regarding the physical association between CYT and DH. The lack of full-length CDH structures has also hampered analysis of the possible mechanisms for ET between CDH and external electron acceptors such as LPMOs.

Here we report the crystal and solution structures of open and closed states of two fungal CDHs and one LPMO. The closed CDH structure reveals a shielded IET pathway from FAD in the DH domain to the haem *b* in the CYT domain. Haem propionate-A in CYT enters the DH active site to interact with four side chains that we refer to as the propionate-docking site on DH. To evaluate whether this closed structure represents the relevant conformational state for productive IET, we performed rational site-directed mutagenesis of selected residues positioned between the FAD cofactor and the haem propionate-A, as well as rapid-kinetics measurements, to probe IET between FAD and haem *b* in the CDH variants. By applying small-angle X-ray scattering to deglycosylated and glycosylated forms of CDH in the absence and presence of an inhibitor, we demonstrated that both the open and closed CDH states are represented in solution. We also show, for the first time, direct and rapid ET between CYT and LPMO, and that DH is unable to transfer electrons to LPMO. Our CDH crystal structures provide a necessary structural platform for further studies on the interaction mechanism between CDH and LPMO during cellulose depolymerization.

## Results

### Crystal structures of the closed and open states of CDH

**Figure 1:**
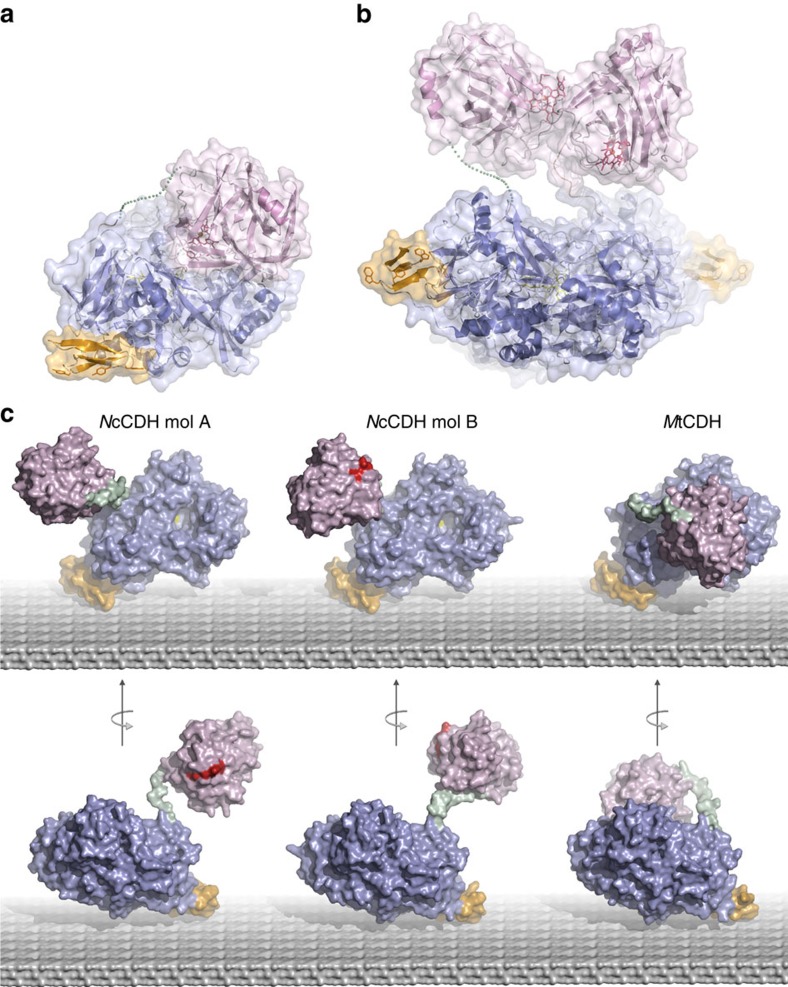
Conformational states of *Mt*CDH and *Nc*CDH. (**a**) *Mt*CDH in the closed state shown as a ribbon drawing with a superimposed semitransparent surface. The missing residues 211–217 in the linker are shown as a green dotted line. Colour-coding: CYT domain (pink), DH domain (blue), CBM (orange), haem *b* (red), FAD (yellow). (**b**) *Nc*CDH with the same colouring scheme as in (**a**) showing the NCS-dimer of the asymmetric unit. The missing residues 206–217 in molecule B are shown as a green dotted line. (**c**) The crystal structure of *Nc*CDH and *Mt*CDH represented by molecular surfaces and modelled on an idealized crystalline cellulose surface. The molecules are oriented to optimize interaction of the cellulose-binding domain (orange) with the cellulose surface, and displayed in two views related by a 180° rotation to visualize the relative positions of the CYT (pink), DH (blue) and linker (green) domains. The two *Nc*CDH molecules A and B of the asymmetric unit have different linker conformations, revealing two unique open states. The haem *b* group in the CYT domain is highlighted in red, and the entrance to the active site where the buried FAD molecule is visible (yellow) is indicated for *Nc*CDH molecule B. For clarity, the missing residues in the linkers of *Mt*CDH and *Nc*CDH have been modelled and *N*-linked glycans omitted.

**Figure 2:**
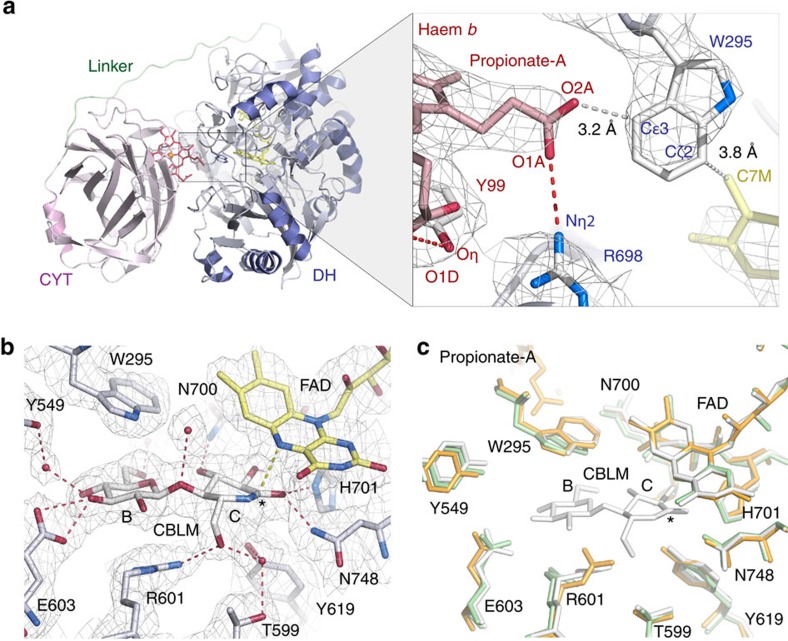
Details of the closed state of *Mt*CDH. (**a**) Ribbon drawing (left) of the closed IET-competent state of *Mt*CDH showing the association of the CYT domain (pink) and the DH domain (blue). The inset (right) highlights the relative orientation of the haem *b* in CYT (red) and the FAD (yellow) in DH (blue) with the 2*F*_0_−*F*_c_ electron density calculated at 3.2 Å and contoured at 0.8*σ*. Dashed red lines represent interactions within hydrogen-bonding distance, and grey dashed lines the edge-to-edge distances for haem *b*-Trp295-FAD. (**b**) Binding of CBLM in the active site of *Mt*DH overlaid by a 2*F*_0_−*F*_c_ electron density contoured at 1.3*σ*. The asterisk denotes the position in CBLM corresponding to the site of oxidation in cellobiose. The binding subsites are named B and C. (**c**) Comparison of the active site in *Mt*CDH (orange), *Mt*DH-CBLM (white) and ligand-free *Mt*DH (green). The catalytic amino acids His701 and Asn748 are positioned at the *re*-side of the flavin.

We screened a range of basidiomycete and ascomycete fungi and ultimately achieved successful crystallization and structure determination of two full-length CDHs from the ascomycetes *Myriococcum thermophilum* (*Mt*CDH) and *Neurospora crassa* (*Nc*CDH) (Fig. 1), and of LPMO_9F_ from *N. crassa*. The crystal structure of *Mt*CDH was determined at 3.2 Å and revealed a closed state in which the CYT domain is docked onto the DH domain in an arrangement that would allow efficient IET from FAD to haem *b* (Fig. 2a). In the closed IET-competent state, the haem *b* propionate-A stretches into the active-site pocket in DH, where the propionate carboxyl group forms an anion–quadrupole interaction with the electropositive edge of the Trp295 benzene ring (Fig. 2a). Propionate-A engages in an ionic interaction with Arg698, which stabilizes the propionate in its ionized state. The haem *b* propionate-D is folded away, and a hydrogen bond to Tyr99 in CYT prevents it from interacting directly with the DH active site. The closest edge-to-edge distance between haem *b* and FAD is 9 Å. This distance is well within the 14-Å limit for efficient electron transfer^27^ and is nearly identical to the haem-FMN distance of 9.7 Å in flavocytochrome *b*_2_, for which rapid IET has been observed^28^.

We observed that crystals of native, full-length *Nc*CDH typically lacked interpretable density for the CYT domains. Data from one platinum-soaked crystal offered better-defined electron density for the CYT domains, which allowed us to model both *Nc*CDH molecules in the asymmetric unit. The overall weak density for the CYT domains suggests significant flexibility in the linker regions. The two non-crystallographically (NCS)-related molecules are present as two ‘open' states with different conformations of the flexible linkers and different relative orientations of the CYT and DH domains (Fig. 1). The haem *b* is fully exposed and accessible in both open-state models.

### The active site in CDH is accessible in the closed state

The active site of CDH has two glucosyl-binding subsites for cellobiose binding, subsite B (for binding site) and C (for catalytic site)^26^,^29^. We determined the crystal structure of *Mt*DH in complex with the substrate analogue cellobiono-1,5-lactam (CBLM). In this complex, Trp295 acts as a platform for the non-reducing end glucosyl unit in subsite B (Fig. 2b). Despite differences in active site side-chain composition (Supplementary Fig. 1), we observed that binding of CBLM in *Mt*DH is nearly identical to that previously reported for the *P. chrysosporium* DH co-crystal structure^29^ (Supplementary Fig. 2). Superimposition of the structures of *Mt*CDH and *Mt*DH-CBLM with ligand-free *Mt*DH revealed that only two active-site side chains differ in conformation: the indole ring of Trp295 tilts slightly ‘upwards' with a maximum ring displacement of 1.4 Å, and Arg601 undergoes a conformational change involving 180° rotations of χ3 and χ4 on ligand binding at site B (Fig. 2c). Comparison of *Mt*CDH and *Mt*DH-CBLM revealed that neither the entry of propionate-A nor the binding of CBLM causes significant changes in the active site. Thus, the substrate and product can be spatially accommodated in the active site while CYT is docked onto DH in the closed state. A channel leads from the surface into the active site of the closed *Mt*CDH molecule. The size of this channel is sufficient (∼11 × 12 Å) to permit substrate entry and product exit while *Mt*CDH remains in the closed IET-competent state (Supplementary Fig. 3).

### Analysis of the FAD–haem *b* interaction by mutagenesis

**Figure 3:**
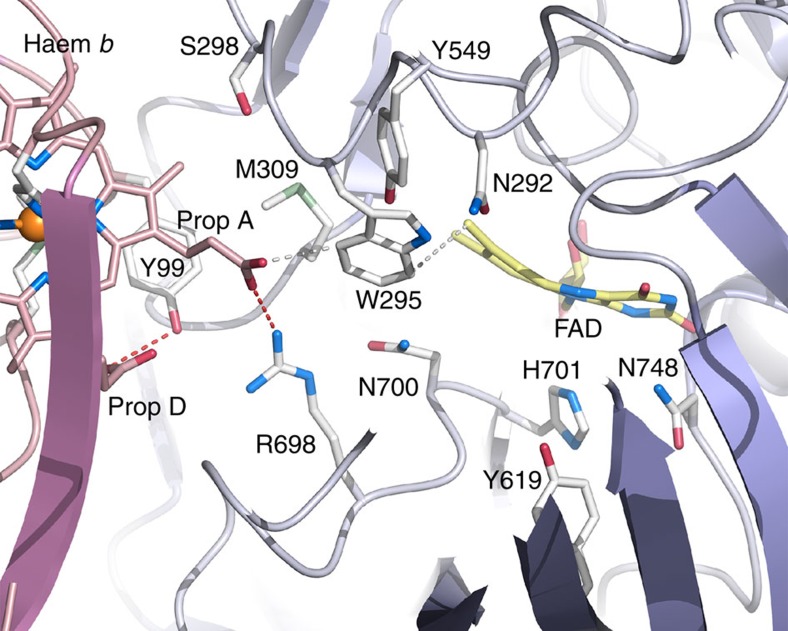
Amino acids in *Mt*CDH targeted for mutagenesis. Close-up of the interface between the cytochrome and dehydrogenase domains in *Mt*CDH showing the side chains targeted for mutagenesis to evaluate the haem *b* propionate-A interactions in the closed state (Trp295, Ser298, Met309 and Arg698) and the substrate-binding region (Asn700, Asn292, Tyr619 and Tyr549).

We mutated positions in the substrate-binding region and at the CYT-DH interface (Fig. 3) to investigate the validity of the closed state of *Mt*CDH for IET. The *Mt*CDH structure indicates that the haem *b* propionate-A in CYT interacts with four side chains in DH at the CYT-DH interface, that is, Trp295, Ser298, Met309 and Arg698, a region that we refer to as the propionate-docking site on DH. In the propionate-docking site, Trp295 performs an important role as a stacking platform for the non-reducing end glucosyl unit of the cellobiose substrate (Fig. 2b,c). In contrast, Ser298, Met309 and Arg698 do not interact with the substrate but with haem propionate-A (Fig. 3). The variants targeting the propionate-docking site included W295A, S298Q, M309A, M309R and R698S. Another set of mutations targeted side chains in the cellobiose-binding region that could potentially affect IET, that is, Asn292, Tyr549, Tyr619 and Asn700, by generating the single mutants N292S, Y549F, Y619Q and N700S.

### Replacement of propionate-interacting residues in DH

The reduction kinetics of FAD and haem *b* indicated that mutations that target the propionate-docking site but not substrate binding (that is, S298Q, M309A, M309R, R698S) have little impact on FAD-reduction rates but large negative effects on haem *b* reduction rates (Table 1). A notable exception is W295A, which shows similar performance as the wild-type enzyme. At the employed high cellobiose concentration, the apparent rate of FAD reduction is not compromised by the W295A mutation. The 12% higher haem *b*-reduction rate of this variant is most likely due to minor structural rearrangements. The barely affected rate of this mutation demonstrates that Trp295 is not essential for IET.

The side chain of Ser298 packs against the haem *b* methyl group (attached to pyrrole ring A), adjacent to propionate-A (Fig. 3). Replacing Ser298 with glutamine leads to a minor decrease of 18% in the FAD-reduction rate, while the haem *b* reduction rate decreases drastically by 88% (Table 1). The decrease in haem *b k*_obs_ for S298Q can be rationalized by steric clashes between the longer glutamine side chain and haem pyrrole A (and with Gln175) in CYT that push propionate-A out from the propionate-docking site on DH. In the wild-type enzyme, Met309 forms van der Waals interactions with the aliphatic carbons of the propionate-A side chain and Tyr99 in CYT, as well as with Trp295, Arg698, Asn700 and the FAD 8-methyl group in DH (Fig. 3). Most, if not all, of these interactions would be eliminated in the M309A mutant, which also exhibits a drastic decrease in the haem *b* reduction rate. Thus, Met309 is important for CYT-DH association, but interpretation of the effects of this mutation is complicated by the threefold reduction in the FAD-reduction rate, which indicates that the mutation has effects beyond the precise association of the functional domains. Another replacement at this position, M309R, maintains the aforementioned interactions with an unperturbed FAD-reduction rate while selectively interfering with propionate-A docking. The loss of IET in this mutant is attributable to the longer arginine side chain, which either pushes CYT away from the propionate-docking site on DH or locks the propionate-A carboxyl group in an ionic interaction. The guanidium group of Arg698 interacts directly with propionate-A, and replacing this side chain with serine abolishes haem *b* reduction without affecting the FAD reduction rate, which is consistent with the observed structure of the closed state of *Mt*CDH.

### Replacement of residues in the substrate-binding region

The side chain of Asn292 is positioned in subsite B, close to Trp295, but does not interact with either CYT or the haem *b* group (Fig. 3). The FAD-reduction rate is increased by 20% for the N292S mutant at the high concentration of cellobiose (25 mM), with a proportional increase in the rate of haem *b* reduction (Table 1). Asn292 has no significant direct effect on the CYT-DH association or IET, which is also in agreement with the closed *Mt*CDH structure. Another residue at the DH active-site entrance is Tyr549 (Fig. 3), which is located in the channel that runs across the CYT-DH interface. Here Tyr549 makes no direct contact with either the CYT domain, haem *b* propionates, or substrate and is therefore not expected to affect either the CYT-DH association or IET. The unperturbed FAD and haem *b* reduction rates of Y549F confirm that the Tyr549 hydroxyl group is not important for IET. The side chain of Tyr619 is located in subsite C in DH, where it likely stabilizes the transition state during cellobiose oxidation^29^. We observed a 2-fold decrease in the *k*_obs_ values for both FAD and haem *b* reduction for Y619Q. The pronounced decrease in the FAD-reduction rate indicates that this variant is catalytically defective, which suggests that Tyr619 has functions other than promoting IET. The side chain of Asn700 packs against the dimethyl benzene nucleus of FAD, where it can form a hydrogen bond with the O3 hydroxyl of the reducing-end glucosyl of cellobiose in subsite C (Figs 2b and 3) and with Arg698. The variant N700S displays a 1.3-fold increase in the FAD-reduction rate and a disproportionally higher 1.8-fold increase in the haem *b* reduction rate. The selective improvement in the IET rate may be due to an increase in the volume of the propionate-docking site caused by the substitution with the smaller serine residue, which may allow Arg698 to move closer to FAD, and consequently, bring propionate-A closer to the flavin.

### Solution structures of CDH

We used small-angle X-ray scattering (SAXS) analysis to investigate the conformational space of CDH in solution using the ensemble-optimization method (EOM), which employs a genetic algorithm for the selection of conformers from pools of randomly generated models^30^,^31^. SAXS was performed on glycosylated and deglycosylated *Mt*CDH and *Nc*CDH in the absence and presence of bound inhibitor (CBLM). Our results demonstrated that all *Mt*CDH and *Nc*CDH samples contain similar subsets of conformers in solution (Supplementary Figs 4 and 5). The conformer most similar to the closed state in the crystal structure of *Mt*CDH is present in all samples (deglycosylated *Mt*CDH cluster 5, deglycosylated *Mt*CDH/CBLM cluster 3, glycosylated *Mt*CDH cluster 1 and glycosylated *Mt*CDH/CBLM cluster 4 in Supplementary Fig. 4). In the presence of inhibitor, the ensemble of glycosylated *Mt*CDH exhibits fewer conformers in solution, with an open state resembling molecule A in the crystal structure of *Nc*CDH as the most populated species (45%) and a closed state resembling the *Mt*CDH crystal structure as the second most populated conformer (27%). Conformers resembling the closed state are also present in all *Nc*CDH samples (deglycosylated *Nc*CDH cluster 8, deglycosylated *Nc*CDH/CBLM cluster 7, glycosylated *Nc*CDH cluster 2 and 3, and glycosylated *Nc*CDH/CBLM cluster 1 and 4 in Supplementary Fig. 5). As with *Mt*CDH, glycosylated *Nc*CDH exhibits an ensemble with fewer conformational clusters in the presence of inhibitor. We also confirmed that glycosylated and deglycosylated *Mt*CDH and *Nc*CDH were present in solution as monomers by performing chemical cross-linking, size-exclusion chromatography and native PAGE (data not shown).

### Crystal structure of *Nc*LPMO_9F_

**Figure 4:**
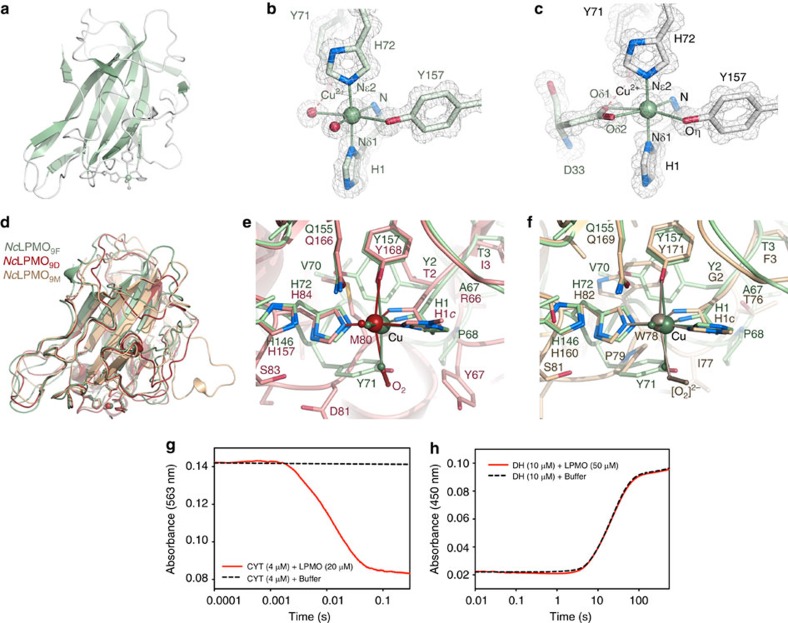
Structure of *Nc*LPMO_9F_ and interaction with *Nc*CYT. (**a**) Overall structure of *Nc*LPMO_9F_. (**b**) Distorted octahedral coordination for the copper in *Nc*LPMO_9F_ molecule A (green). The three nitrogen ligands provided by His1 and His72 are referred to as the *histidine brace*^10^. Coordination is satisfied by two water ligands (see Supplementary Fig. 6 for details). Overlay with 2*F*_0_−*F*_c_ electron density (1.5*σ*). (**c**) Cu(II) coordination in *Nc*LPMO_9F_ molecule B (white). Octahedral coordination is satisfied by replacing the water ligands in (**b**) by two oxygen atoms from Asp33 (green) in the NCS-related A molecule. (**d**) Structural overlay of *N. crassa* LPMO_9F_ (green) with *N. crassa* PMO-2 (red; PDB code 4EIR^23^) and PMO-3 (beige; PDB code 4EIS^23^). (**e**) Comparison of the copper centres in *Nc*LPMO_9F_ (green) and PMO-2. (**f**) Comparison of the copper centres in *Nc*LPMO_9F_ (green) and PMO-3. The residue H1*c* represents a methylated N-terminal histidine. (**g**) Interaction between *Nc*CYT and *Nc*LPMO_9F_ displayed as an averaged kinetic trace (full line). The calculated haem re-oxidation rate from five repeated experiments (*k*_obs_) is 67.2±2.3 s^–1^. For comparison, the haem re-oxidation by oxygen is shown as a dashed line. (**h**) The re-oxidation rates of the FAD cofactor of the *Nc*CDH dehydrogenase domain (10 μM) remains unchanged in the presence (0.0442 +/−0.0003, s^–1^) and absence (0.0444 +/− 0.0006, s^–1^) of 50 μM LPMO, showing that no reduction of the *Nc*LPMO_9F_ Cu(II) centre takes place. The observed low rates are the result of a re-oxidation reaction with dissolved oxygen in the buffer.

*Nc*LPMO_9F_ primarily attacks crystalline cellulose and promotes, in combination with cellulases, faster and more complete surface degradation^32^. The structure of *Nc*LPMO_9F_ demonstrates that this LPMO shares the essential features of fungal LPMOs, including a β-sandwich fold and a catalytic surface-exposed copper centre (Fig. 4a) in which the copper is coordinated by the ligands His1, His72 and Tyr157 (Fig. 4b,c). Fungal LPMOs are typically post-translationally modified by protein glycosylation and methylation at the N-terminal histidine (the function of this methylation is unknown). We determined that *Nc*LPMO_9F_ is expressed in *Pichia pastoris* as a 214-residue non-glycosylated protein. As observed for the fungal LPMO GH61D from *Phanerochaete chrysosporium*^33^, the N-terminal histidine in *Nc*LPMO_9F_ is not methylated, in contrast to the methylation of other *N. crassa* LPMOs expressed using the natural fungal host^15^,^23^. Both NCS molecules in the NcLPMO_9F_ crystal have copper ligated by four protein atoms in tetrahedral coordination and exhibit distorted octahedral coordination of the copper with *mer*-[MA_3_B_3_] geometry together with external ligands (Fig. 4b,c; Supplementary Fig. 6). In molecule A, two water molecules, one axial and one equatorial, satisfy the octahedral sphere (Fig. 4b; Supplementary Fig. 6a). In molecule B, both water molecules are replaced by the carboxylate oxygen atoms of Asp33 from molecule A (Fig. 4c; Supplementary Fig. 6b), which is similar to the coordination sphere observed in *Streptomyces coelicolor Sc*LPMO_10B_ where two oxygen atoms in an acetate molecule occupy the fourth equatorial and axial position on the solvent-facing side of Cu(II)^34^. These observations emphasize the possibility of the 4-coordinate Cu(II) in *Nc*LPMO_9F_ to accept one axial, and possibly also a fourth equatorial ligand (water or other).

The crystal structures of *N. crassa Nc*LPMO_9D_ (PMO-2; PDB code 4EIR^23^; NCU01050; UniProt Q1K8B6) and *Nc*LPMO_9M_ (PMO-3; PDB code 4EIS^23^; NCU07898; UniProt Q7SA19) have been determined at high resolution. While the overall fold of *Nc*LPMO_9F_ is very similar to those of *Nc*LPMO_9D_ (r.m.s.d. 1.45 Å for 205 Cα positions) and *Nc*LPMO_9M_ (r.m.s.d. 1.35 Å for 189 Cα positions), significant conformational changes are apparent in loop regions, including loops flanking the copper-binding site (Fig. 4d). In contrast to *Nc*LPMO_9F_, *Nc*LPMO_9D_ and *Nc*LPMO_9M_ are glycosylated and have a methylated N-terminal histidine (Fig. 4e,f). Furthermore, the axial ligand occupied by water or Asp33 Oδ1 in *Nc*LPMO_9F_ (Fig. 4b,c; Supplementary Fig. 6) is modelled as superoxide and peroxide in *Nc*LPMO_9D_ (Fig. 4e) and *Nc*LPMO_9M_ (Fig. 4f), respectively. Beyond the copper ligands, the amino-acid context differs in *Nc*LPMO_9F_ compared with *Nc*LPMO_9D_ and *Nc*LPMO_9M_ (Supplementary Fig. 7).

### *Nc*CYT but not *Nc*DH transfers electrons to *Nc*LPMO_9F_

Next we performed rapid-kinetics experiments to confirm that direct ET occurs between *Nc*CYT and *Nc*LPMO_9F_ (Fig. 4g). We observed very rapid *Nc*CYT-to-*Nc*LPMO_9F_ ET with a haem *b*-*k*_obs_ value of 67.2±2.3 s^–1^. The re-oxidation rate of *Nc*CYT by oxygen was 100,000-fold slower (0.0007, s^−1^) than the rate for *Nc*CYT-to-*Nc*LPMO_9F_ ET, making the oxygen reaction negligible. To further rule out reduction of the *Nc*LPMO_9F_ copper centre by the FAD cofactor in the *Nc*CDH dehydrogenase, we performed a second stopped-flow experiment, which demonstrated conclusively that the *Nc*DH domain alone is unable to reduce Cu(II) in *Nc*LPMO_9F_ (Fig. 4h). Our results demonstrate unequivocally that, in the *N. crassa* CDH IIA-LPMO_9F_ pair, the reduced haem *b* alone acts as the reductant of the *Nc*LPMO_9F_ copper centre *in vitro*. The high ET rate observed in the study provides compelling evidence for LPMO as a physiologically relevant electron acceptor for the haem *b* cytochrome.

To test the interaction of the *Nc*CDH-*Nc*LPMO_9F_ pair, we performed automatic high ambiguity-driven biomolecular docking (*HADDOCK*). The program consistently returned interaction models where the haem *b* propionate-A was docked close to the copper in *Nc*LPMO_9F_. The docking of solvated proteins does not consider the constrained accessibility of the copper site in cellulose-bound LPMO; however, in solution and when freely accessible, the copper site appears to be a favourable docking site for the haem *b* propionate (Supplementary Fig. 8). Because no information concerning the precise copper-haem coordination geometry had been provided to the program, the precise details of the automatically generated CYT-LPMO interaction models cannot be evaluated; however, the interaction energies are favourable.

## Discussion

The crystal and solution structures of two closely related class-II CDHs revealed pronounced flexibility of the linker between the CYT and DH domains to allow the efficient association of the haem *b* with both the FAD electron donor (closed state) and external protein electron acceptors such as LPMO (open states). Using SAXS, we demonstrated that the conformers observed in the crystal structures are also present in solution and performed comprehensive mapping of accessible CDH conformers, including the closed state and a range of partially closed and open states. Moreover, our data demonstrate that similar CDH conformers are possible for glycosylated and deglycosylated samples and that the presence of an inhibitor reduces the number of accessible conformers in solution.

The crystal structure of the closed state of *Mt*CDH offers a favourable association mode between the CYT and DH domains to permit efficient IET. The validity of the closed state is further emphasized by the drastically decreased IET rates of *Mt*CDH variants in which the interactions between haem *b* propionate-A and DH at the CYT-DH interface have been disabled. Whereas the IET rates were diminished for mutations targeting the propionate-docking site, we observed no systematic effects on IET rates for the mutations targeting side chains in the substrate-binding region occupying the space between the flavin and propionate-A. These results allow us to assign a fundamental role to the haem *b* propionate-A in the IET mechanism. Haem propionate groups actively participate in ET events. For example, the use of mixed quantum mechanical/molecular mechanics calculations has provided direct evidence for the active involvement of haem-propionate groups in the ET pathway of ascorbate peroxidase and di-haem-*c* cytochrome *c* peroxidase^35^. Our results, together with the low sequence conservation between ascomycete and basidiomycete CDHs (of the mutated residues, only Tyr619, Asn700 and Tyr549 are conserved in *Pc*CDH; Supplementary Fig. 1), emphasize that efficient IET does not depend intimately on any one specific type of side chain but on the ability of CYT to associate with DH in a manner in which the relative position and distance between the haem propionate and FAD ensure IET.

In contrast to the intracellular enzyme flavocytochrome *b*_2_, CDH is an extracellular flavocytochrome. A variety of two-electron acceptors are generated during lignocellulose decomposition, for example, lignin-derived quinone compounds that would react rapidly with reduced FAD. How is specific FAD-haem *b* IET ensured in this extracellular environment containing electron scavengers? If CYT docks with DH before substrate binding and remains docked during IET (and possibly after product departure), the IET path would be shielded both by the CYT-DH interface and by the bound product, thus preventing electron scavengers from accessing the reduced FAD before the haem *b* is reduced and CYT is released. Indeed, various free one-electron acceptors such as Fe(III) and Cu(II) complexes are present in wood and could scavenge electrons from reduced CYT upon release from DH. The surfaces near the channel entrance in *Mt*CDH are rich in negatively charged residues that do not participate in complex formation (Supplementary Fig. 9a). It is tempting to speculate that their function may be to guard the entrance to allow carbohydrates to enter and exit, while metal ions are chelated and negatively charged molecular species are repelled. Overall, *Nc*CDH has fewer negative surface residues but displays a similar trend of negative charges clustering near the channel entrance (Supplementary Fig. 9b).

In this work, we demonstrate that only the CYT haem *b* of *Nc*CDH can transfer electrons to *Nc*LPMO_9F_ and that DH alone is not an electron donor for LPMO. We expect this ET to manifest as a physical but not necessarily strong or long-lived protein complex. On the basis of global sequence conservation within the LPMO family, a conserved surface patch centred on ^218^Pro-Gly-Pro^220^ (numbering of *Nc*PMO_9M_) has been suggested as an interaction site for CDH^23^. The patch is positioned 21 Å from the copper centre, and its involvement assumes long-range electron tunnelling through the protein to reach the site of reduction. Such a scenario seems mechanistically reasonable for LPMO where, during catalysis, the copper-site is expected to be oriented towards the cellulose surface and therefore is inaccessible for a direct interaction with the redox centre in CYT. It is therefore surprising to find no conserved complementary surfaces on CYT. Rather, the haem *b* propionate-A, which protrudes from the CYT surface and restricts surface complementarity between CDH and LPMO at the proposed interaction site, is the only absolutely conserved surface feature among different CDHs. Because CDH has to form a CYT–DH IET complex and has been observed to transfer electrons to a variety of LPMOs with different surface properties and even from different organisms, it is not surprising that the interacting interfaces display an overall low degree of surface complementarity. The results from ambiguity-driven biomolecular protein-protein docking suggest an alternative binding mode for LPMO when not bound to cellulose where the copper-binding surface provides the most favourable interaction site for CYT. However, it is not possible to evaluate the significance of such an interaction by automated molecular docking alone and mutational studies are needed to elucidate the *in vivo* interaction site.

At present, two principal LPMO mechanisms for glycosidic bond cleavage have been proposed. The first mechanism involves sequential ET^17^, in which an initial electron reduces Cu(II) to Cu(I), leading to the formation of a Cu(II)-superoxo complex. A second electron is required after hydrogen abstraction from the substrate to cleave the O-O bond in the Cu(II)-hydroperoxo species, releasing water and generating a Cu(II)-oxo radical that couples with the substrate radical and leads to the hydroxylation of the substrate. The second mechanism, which was suggested based on quantum mechanical calculations, proposes C-H oxidation by a Cu(II)-oxyl mechanism^24^ and argues that a Cu(II)–oxyl complex is more reactive than the Cu(II)–superoxo complex and that the Cu(II)–oxyl species has a lower overall activation barrier than the Cu(II)–superoxo species. This model hypothesizes two electron transfer events in series before LPMO reacts with cellulose. In both cases, hydroxylation at C1 or C4 destabilizes the glycosidic bond, and an elimination reaction leads to bond cleavage. Both models are compatible with one-electron transfer from CYT to LPMO, but the precise structural and mechanistic details of the CYT-LPMO interaction need further investigation.

In this work, we have presented for the first time the structural basis for electron transfer between FAD and haem *b* in CDH, and shown that the cytochrome alone is responsible for rapid electron transfer to LPMO. Our results provide the structural foundation towards a full molecular understanding of the role of the CDH-LPMO system in oxidative cellulose degradation by fungi.

## Methods

### Enzyme production

Cloning of all genes has been reported: *Myriococcum thermophilum* CDH^36^ (*Mt*CDH IIA; gene *cdh*; UniProt A9XK88; the DH domain belongs to CAZy family AA3_1 and the CYT domain to CAZy family AA8 (ref. 37)), *Neurospora crassa* CDH IIA^21^ (*Nc*CDH; gene *cdh-1*; locus tag NCU00206; UniProt Q7RXM0; the DH domain belongs to CAZy family AA3_1 and the CYT domain to CAZy family AA8 (ref. 37)) and *Neurospora crassa* LPMO *Nc*LPMO_9F_ (ref. 38) (gene *gh61-6*; locus tag NCU03328; UniProt Q1K4Q1; CAZy family AA9).

The *Mt*CDH variants were generated by a two-step mutagenesis approach using PCR and *Dpn*I. The replacements included N292S, W295A, S298Q, M309A, M309R, Y549F, Y619Q, R698S and N700S. The mutations were confirmed by sequencing. Fed-batch fermentations of *Pichia pastoris* X-33 transformants were performed in a 7-litre bioreactor (MBR, Switzerland) with 3 litre starting volume following the *Pichia* Fermentation Process Guidelines of Invitrogen. After depletion of glycerol in the batch medium a 12-h fed-batch phase was started with a constant feed of 50% glycerol containing 12 ml l^–1^ PTM_1_ trace salts to increase biomass. For induction the feed was switched to pure methanol containing 12 ml l^–1^ PTM_1_ trace salts and the cultivation temperature was reduced from 30 to 25 °C. At the time the culture fully adapted to methanol the feed rate was automatically adjusted to keep the dissolved oxygen saturation constant at 4% at a constant air supply of 6 l min^–1^ and a stirrer tip speed of 2.95 m s^–1^. Samples were taken regularly and wet biomass, protein concentration and enzyme activity were measured. Fermentation broths were harvested by centrifugation before the expression of the targeted enzymes stagnated. Wild type and mutant CDHs were expressed in concentrations from 50 to 400 mg l^–1^, and the protein concentration of *Nc*LPMO_9F_ in the culture supernatant was 750 mg l^–1^. Additional details have been reported elsewhere^36^,^38^,^39^,^40^.

### Enzyme purification

*Nc*DH, *Nc*CYT, *Nc*CDH, *Mt*DH and *Mt*CDH variants were purified by a two-step chromatographic procedure starting with hydrophobic interaction chromatography using PHE-Sepharose FF (all chromatographic equipment and materials from GE Healthcare Biosciences). Proteins were applied in a 50 mM sodium acetate buffer (pH 5.5) containing 20% ammonium sulfate (saturation) and eluted by a linear gradient against the same buffer without ammonium sulfate. Fractions containing the target enzyme were pooled and diafiltered with a 50 mM sodium acetate buffer (pH 5.5) using a hollow fibre cross-flow module (Microza UF module SLP-1053, 10 kDa cut-off, Pall Corporation). Concentrated CDH pools were applied to a column packed with Source 15Q material equilibrated with 50 mM sodium acetate buffer (pH 5.5) and eluted within a linear salt gradient from 0 to 1 M NaCl within 10 column volumes.

*Nc*LPMO_9F_ was purified by a three-step chromatographic procedure starting with hydrophobic interaction chromatography using PHE-Sepharose FF. The protein was loaded in 25 mM sodium acetate buffer (pH 5.0) containing 30% ammonium sulfate (saturation), and eluted by a linear gradient. Fractions containing the enzyme were pooled and diafiltered with a 20 mM Tris-HCl buffer (pH 8.0) using a hollow fibre cross-flow module. After reaching a conductivity below 1.4 mS cm^–1^ the pool was applied to a column packed with Source 15Q. The flow-through contained *Nc*LPMO_9F_ and was concentrated and further purified with size-exclusion chromatography using a Superdex 75 column equilibrated with 20 mM Tris-HCl buffer (pH 8.0). Fractions containing pure *Nc*LPMO_9F_ were pooled, concentrated to a final concentration of 6.5 mg ml^–1^ and stored at 4 °C.

### Deglycosylation and proteolytic cleavage of CDHs

The purified CDHs were treated at 30 °C for 18 h with 3,200 U ml^–1^ α-1,2/3-mannosidase and 50,000 U ml^–1^ endoglycosidase H_f_ (New England Biolabs, Ipswich, MA, USA) in 50 mM sodium acetate buffer (pH 5.5) containing 5 mM ZnCl_2_ to obtain 10 mg ml^–1^ deglycosylated enzyme. To remove the glycosidases, column chromatography with Source 15Q was repeated as described above and the fractions containing pure CDH were pooled, diafiltered to 50 mM sodium acetate buffer (pH 5.5) and stored at 4 °C. Proteolytic cleavage in the linker of CDH was performed to obtain the individual DH and CYT domains. To this end, 40 μl of papain (10 mg ml^–1^) was incubated at 25 °C for 1 h with 100 μl of activation buffer containing 2 mM EDTA and 2 mM dithiothreitol in 100 mM sodium phosphate (pH 7.0). CDH (final concentration 10 mg ml^–1^) was digested in a reaction mix containing 140 μl per ml of the activated papain solution and 1 M sodium acetate (pH 5.0) at 25 °C for 4 h. The domains were separated from the residual intact CDH by column chromatography using a strong anion exchanger (Mono Q). The sample was diafiltered to 20 mM Tris-HCl (pH 8.0), loaded on the column and eluted by a linear NaCl gradient. Fractions containing the dehydrogenase and the cytochrome domain were pooled, concentrated and stored at 4 °C for further use.

### Kinetic characterization of *Mt*CDH wild type and variants

Pre-steady-state measurements were carried out in a stopped-flow spectrophotometer (Applied Photophysics SX 20, Leatherhead, UK) at 30 °C. The concentration of enzymes was determined by their molar absorption coefficients (*Nc*CDH *ɛ*_420_=100 mM^–1^ cm^–1^, *Nc*DH *ɛ*_450_=11.3 mM^–1^ cm^–1^, *Nc*LPMO_9F_ *ɛ*_280_=46.9 mM^–1^ cm^–1^). The FAD and haem *b* reduction rates in CDH were measured at 449 and 563 nm, respectively, by reducing 5 μM CDH with 25 mM cellobiose (final concentrations) in 50 mM sodium citrate buffer pH 5.0.

### Electron transfer *Nc*CYT–*Nc*LPMO_9F_ and *Nc*DH–*Nc*LPMO_9F_

The same technique was used for measuring the ET between *Nc*CYT or *Nc*DH and *Nc*LPMO_9F_. *Nc*CYT was partially reduced with sodium dithionite (15 mM stock solution) by following the spectra in a diode array photometer. By partial reduction, excess of reductant was prevented. The 50% reduced *Nc*CYT was immediately transferred to a stopped-flow spectrophotometer and mixed with *Nc*LPMO_9F_ in single mode. Re-oxidation of the α-band of *Nc*CYT haem *b* was recorded at 563 nm. Concentrations in the cell were 4 μM reduced *Nc*CYT and 20 μM *Nc*LPMO_9F_ in 50 mM sodium citrate buffer pH 5.0. The observed rates were fitted to a single exponential function. Reduction and re-oxidation of the FAD cofactor in DH was followed at 450 nm. Experiments were performed in sequential mode by mixing 40 μM *Nc*DH with 100 μM cellobiose to reduce the enzyme. The reaction was held in an ageing loop for 75 s until ∼10% of the *Nc*DH was re-oxidized. The partly re-oxidized *Nc*DH was shot against buffer or *Nc*LPMO_9F_. Final concentrations in the measuring cell were 10 μM *Nc*DH and 50 μM *Nc*LPMO_9F_. The rates were calculated from an exponential fit.

### Crystallization, structure determination and model refinement

Data collection and refinement statistics are given in Table 2. All protein crystallization was performed using the sitting-drop vapour diffusion method at room temperature. Image processing and data scaling were performed with the *XDS* package^41^. When applicable, molecular replacement (MR) was performed using *PHASER*^42^ as implemented in the *PHENIX* suite^43^, and unless otherwise stated experimental phasing was performed with *autoSHARP*^44^. Manual model building and correction was performed with the programs *O*^45^ and *COOT*^46^. Experimental details for each protein are given below. For models refined at lower resolution, the resolution cutoff for refinement was guided by the *CC*(1/2) values, that is, the percentage of correlation between intensities from random half data sets^47^.

*MtCYT (MtCDH cytochrome domain)*. Crystals of deglycosylated *Mt*CYT were obtained by mixing equal volumes of protein solution (21 mg ml^–1^ in 20 mM HEPES pH 7.5) and a solution containing 0.1 M Tris-HCl (pH 8.4), 0.2 M MgCl_2_, 30% (w/v) polyethylene glycol 4,000. The crystals belong to space group *P*2_1_ with two molecules in the asymmetric unit. A model for molecular replacement (MR) was generated automatically using the *BALBES* server^48^. MR calculations were performed with *PHASER*, and a model was traced and built automatically using data to 1.4 Å resolution with the *warpNtrace* function in *ARP/wARP*^49^. The model was refined at 1.4 Å resolution with *PHENIX*, using the maximum-likelihood target and including refinement of coordinates, real-space refinement and refinement of individual anisotropic temperature factors (Table 2; Supplementary Fig. 10a). The refined model contains: two protein chains, A and B, each composed of residues 1–208 (corresponding to residues 22–229 in UniProt A9XK88); one type-*b* protoheme IX group per protein chain; protein glycosylation (one *N*-acetyl glucosamine, NAG, residue attached to Asn119, and four *O*-linked mannose residues attached to Ser195, Thr197, Thr204 and Thr206, respectively, per protein chain); one magnesium ion bound to protein chain A and two magnesium ions to chain B; and 350 water molecules.

*MtDH (MtCDH dehydrogenase domain)*. Non-deglycosylated *Mt*DH crystallized from mixing equal volumes of a solution containing 0.1 M sodium acetate (pH 4.6), 0.1 M CdCl_2_, 18% (w/v) polyethylene glycol monomethyl ether 550 with protein (58 mg ml^–1^ in 20 mM HEPES pH 7.5). The crystals belong to space group *P*6_3_ with one molecule in the asymmetric unit. Cryo protection was performed by equilibration in a solution containing the crystallization liquor, but with 30% (w/v) polyethylene glycol monomethyl ether 550. Data were collected on a native crystal, as well as two heavy-atom derivatives, lead acetate and mercury acetate. The heavy atoms were added as powder to the cryo-protection solution and the crystals left to equilibrate in the presence of heavy atom for 1 min before being vitrified in liquid nitrogen. For the complex of *Mt*DH with the inhibitor 5-amino-5-deoxy-cellobiono-1,5-lactam (CBLM) a crystal was used that resulted from equal volumes of 58 mg ml^–1^ protein in 20 mM HEPES pH 7.5 and 0.1 M sodium acetate (pH 4.6), 0.1 M CdCl_2_, 15% (w/v) polyethylene glycol monomethyl ether 2000. The crystal was equilibrated in a solution containing 0.1 M sodium acetate (pH 4.6), 0.1 M CdCl_2_, 30% (w/v) polyethylene glycol monomethyl ether 2000 and 2 mM cellobionolactam inhibitor.

The *Mt*DH structure was determined using multiple isomorphous replacement with anomalous scattering (MIRAS) with lead acetate and mercury acetate using *autoSHARP*^44^. *SHELXD*^50^ in *autoSHARP* was used to locate the heavy-atom positions, and *SHARP*^44^ to refine heavy-atom positions and calculate phases. Density modification using a solvent content of 66% was performed with *SOLOMON* in *autoSHARP* and *DM* in the *CCP4* suite^51^. The final MIRAS phases to 3 Å were of high quality and during the process of iterative model building and refinement, averaged structure factors from the derivatives where the heavy-atom contribution had been removed (*F*_av_) were used together with Hendrickson–Lattman coefficients to allow phase combination of experimental MIRAS phases with partial model phases (*F*_c_) in *REFMAC5*^52^ to improve refinement, phases and *2F*_0_*–F*_c_ electron density quality. The structure of the *Mt*DH–CBLM complex was determined by MR with *PHASER* using the *Mt*DH model as search probe.

The software *PHENIX* was used to refine the *Mt*DH and *Mt*DH-CBLM models at 2.7 and 2.4 Å resolution, respectively (Table 2; Supplementary Fig. 10b,c). Refinement incorporated the maximum-likelihood target, refinement of coordinates, real-space refinement, refinement of individual isotropic temperature factors and translation-libration-screw (TLS) refinement using TLS groups derived by the *PHENIX* software (five groups for *Mt*DH and six groups for *Mt*DH–CBLM). The refined *Mt*DH model contains: one protein chain (A) composed of residues 223–807 (corresponding to residues 244–828 in UniProt A9XK88); one non-covalently bound FAD molecule; protein glycosylation (two NAG residues attached to Asn400, two NAG residues at Asn437, and one NAG at Asn516); 9 cadmium ions; and no water molecules. The resulting *Mt*DH-CBLM model contains: one protein chain (A) composed of residues 223–807; one non-covalently bound FAD molecule; protein glycosylation (two NAG residues attached to Asn400 and two NAG residues at Asn437); one CBLM molecule; two cadmium ions; and 182 water molecules.

*MtCDH*. Deglycosylated *Mt*CDH was crystallized from mixing equal volumes of 0.1 M MES-OH (pH 6.5), 0.1 M ammonium sulfate, 0.4 M sodium formate, 30% (w/v) polyethylene glycol monomethyl ether 5,000 with protein (84 mg ml^–1^ in 20 mM sodium acetate pH 5.5). The crystals were in point group 422, with one molecule in the asymmetric unit. The space group was deduced at the structure determination stage. The individual *Mt*CYT and *Mt*DH domains, refined at 1.4 Å and 3.0 Å resolution, respectively, and *Mt*CDH data (4.5–15 Å) were used for MR calculations using *PHASER*. All possible PG422 enantiomorphs were tested, returning a clear solution only in space group *P*4_3_2_1_2. The *Mt*CDH model was refined against the maximum-likelihood target in *PHENIX* at 3.2 Å resolution (Table 2; Supplementary Fig. 11a) using a refinement protocol including *XYZ* coordinate refinement, refinement of grouped isotropic temperature factors (two groups per residue). The refined *Mt*CDH model contains one protein chain (A) composed of residues 1–807 (corresponding to residues 22–828 in UniProt A9XK88; 1–21 belong to the signal peptide) with residues 211–217 of the linker missing; one type-*b* protoheme IX group; one non-covalently bound FAD molecule; and protein glycosylation (one NAG residue attached to Asn400, Asn437, Asn516 and Asn678; one mannose residue attached to Ser195, Thr197, Thr204, Thr206 and Thr226).

*NcCDH*. Deglycosylated *Nc*CDH was crystallized from mixing equal volumes of 0.1 M MES-OH (pH 6.5), 1.5 M magnesium sulfate, 0.02 M lithium sulfate with protein (36 mg ml^–1^ in 20 mM sodium acetate pH 5.5). Crystals were equilibrated in the crystallization solution supplemented with 50% saturated lithium sulfate before being vitrified in liquid nitrogen. The crystals were in space group *P*2_1_2_1_2_1_ with two molecules in the asymmetric unit. The platinum derivative was produced by adding K_2_Pt(CN)4 powder to a drop containing the crystal in its mother liquor. Initial phasing was performed using Pt-SAD in *autoSHARP*. Phases were improved by density modification with *SOLOMON* using a solvent content of 61.3% to 2.9 Å resolution. To facilitate model building, the *Mt*CYT and *Mt*DH domains were placed in the *Nc*CDH unit cell using MR calculations with *PHASER*. Model re-building was guided by MR- and SAD-phased electron-density maps. Both *Nc*CDH molecules in the asymmetric unit are in the open conformation with dissociated CYT and DH domains. The inherent flexibility of the linker connecting the CYT and DH domains resulted in different NCS symmetry for the CYT and DH domain pairs in the asymmetric unit, and partly disordered linker regions. The *Nc*CDH model was refined using the maximum-likelihood target in *PHENIX* at 2.9 Å resolution (Table 2; Supplementary Fig. 11b) using a refinement protocol including *XYZ* coordinate refinement, refinement of individual isotropic temperature factors and TLS refinement (six groups). The refined model contains two protein chains (A and B) composed of residues 2–806 (corresponding to residues 25–829 in UniProt Q7RXM0; 1–23 belongs to the signal peptide) with residues 206–217 in the linker missing in protein chain B; one type-*b* protoheme IX group and one non-covalently bound FAD molecule per protein chain; protein glycosylation (Asn119, Asn278, Asn400, Asn471, Asn515, Asn541 and Asn555 each have one *N*-linked NAG residue); one mannose residue attached to Thr222 and Thr226; and 10 platinum atoms bound per protein chain.

**Nc*LPMO_9F_*. *Nc*LPMO_9F_ is natively non-glycosylated and contains one copper centre. The copper content of purified *Nc*LPMO_9F_ was analysed by inductively coupled plasma atomic emission spectroscopy, ICP-AES, and sector field inductively coupled plasma mass spectroscopy, ICP-SMS (ALS Scandinavia AB). Crystals of *Nc*LPMO_9F_ were produced by mixing 0.1 μl of protein (30 mg ml^–1^ in 50 mM Tris pH 8.0) with 0.2 μl of reservoir solution containing 0.2 M ammonium nitrate and 20% (w/v) polyethylene glycol 3350. The crystals belong to space group *P*2_1_2_1_2 with two molecules in the asymmetric unit. Structure determination was performed by taking advantage of the copper centre for Cu-SAD using the *AutoSol* and *AutoBuild* modules in *PHENIX*. The best SAD solution had an estimated map CC × 100 of 29.0 +/− 28.3. Density modification and model building were performed iteratively by *RESOLVE*^53^ using a default solvent content of 50%, yielding a model with an *R* factor of 0.28 and a correlation of local RMS density of 0.75. The *Nc*LPMO_9F_ model was refined using data at 1.1 Å resolution (Table 2; Supplementary Fig. 11c) with *PHENIX*, using the maximum-likelihood target and including refinement of *XYZ* coordinates, real-space refinement, refinement of individual anisotropic temperature factors, and riding hydrogen atoms. The refined *Nc*LPMO_9F_ model contains two protein chains (A and B) each composed of residues 1–214 (corresponding to residues 18–231 in UniProt Q1K4Q1; 1–17 constitutes a signal peptide); one copper per protein chain; one nitrate molecule in chain B, and 838 water molecules. Modelling of *Nc*CDH-*Nc*LPMO_9F_ was performed by manual docking of *Nc*CDH CYT such that the haem *b* propionate-A carboxyl group superimposed with that of the copper-coordinating Asp33 in NCS molecule A of *Nc*LPMO_9F_.

### SAXS data collection and analysis

Samples of deglycosylated and glycosylated *Mt*CDH and *Nc*CDH were prepared at the concentrations 1, 2.5 and 10 mg ml^–1^ in 50 mM sodium acetate (pH 4.5), and in the absence or presence of 1 mM CBLM. Data were collected through mail-in-SAXS on the 12.3.1 SIBYLS beamline at the Advanced Light Source, Lawrence Berkeley National Laboratory^54^,^55^, and at the MAX II SAXS beamline *I*911-SAXS^56^ at MAX IV Laboratory, Lund, Sweden. Data were processed using *PRIMUS*^57^ in the *ATSAS* suite^31^. The ensemble optimization modelling method^30^,^31^ was used to generate the most populated clusters of conformers in an ensemble that best fits the scattering data. The crystal structures of CYT and DH were input as domains and 10,000 models with different domain orientations and automatically modelled linkers were generated within the EOM 2.0 framework^58^.

### Chemical cross-linking

Deglycosylated and glycosylated *Mt*CDH and *Nc*CDH were diluted to a final concentration of 0.5–1 mg ml^–1^ with 50 mM HEPES buffer (pH 7.5). The cross-linking was performed by adding glutaraldehyde at a final concentration of 0.5% (v/v) to the diluted protein solutions. The effect of cross-linking was tested in the absence and in the presence of 1 mM EDTA. Samples were taken before adding the cross-linker as well as at *t*=5, 15, 30 and 60 min after adding the cross-linker. One molar Tris buffer pH 7.5 was immediately added to the samples to neutralize the cross-linker and stop the reaction, after which the samples were mixed with sample buffer and reducing agents (50 mM dithiothreitol), heated for 5 min at 75 °C, and loaded on 3–8% NuPAGE Tris Acetate gels (Life technologies).

### Automated molecular docking of *Nc*CDH-CYT and *Nc*LPMO_9F_

We performed automated molecular docking of *Nc*LPMO_9F_ and *Nc*CDH with the program *HADDOCK* 2.0 (http://haddock.science.uu.nl/services/HADDOCK/haddock.php^59^,^60^,^61^). The program requires suggestions for interacting regions, and two hypotheses were tested. One protocol evaluated an interaction between the previously proposed surface patch on LPMO^23^ and the haem in CYT, and another protocol tested an interaction between the copper-binding surface of LPMO and haem. Only the structures of the *Nc*CYT domain and *Nc*LPMO_9F_ were used.

### Illustrations

Figures were produced using the program PyMOL (DeLano, www.pymol.org).

## Additional information

**How to cite this article:** Tan, T.-C. *et al*. Structural basis for cellobiose dehydrogenase action during oxidative cellulose degradation. *Nat. Commun.* 6:7542 doi: 10.1038/ncomms8542 (2015).

## Supplementary Material

Supplementary Information Supplementary Figures 1-11

## Figures and Tables

**Table 1:** FAD and haem *b* reduction kinetics of *Mt*CDH wild type and variants.

***Mt*****CDH variant**	**FAD,** ***k***_**obs**_ **(s**^−1^)	**haem** ***b***, ***k***_**obs**_ **(s**^−1^)
Wild type	19.9±1.1	0.76±0.01
		
*Propionate-A interaction*		
W295A	21.7±2.3	0.86±0.01
S298Q	16.4±0.2	0.090±0.007
M309A	6.9±0.6	0.056±0.001
M309R	15.4±0.9	0.088±0.006
R698S	19.2±1.7	0.011±0.001
		
*Substrate-binding region*		
N292S	23.9±0.3	0.95±0.02
Y549F	18.8±1.1	0.76±0.02
Y619Q	11.0±2.1	0.38±0.01
N700S	26.1±1.1	1.37±0.02

**Table 2:** Data collection, phasing and refinement statistics.

	***Mt*****CYT**	***Mt*****DH**	***Mt*****DH Hg-MIR**	***Mt*****DH Pb-MIR**	***Mt*****DH CBLM**	***Mt*****CDH**	***Nc*****CDH Pt-SAD**	***Nc*****LPMO**_**9F**_	***Nc*****LPMO**_**9F**_ **Cu-SAD**
*Data collection*									
Space group	*P*2_1_	*P*6_3_	*P*6_3_	*P*6_3_	*P*6_3_	*P*4_3_2_1_2	*P*2_1_2_1_2_1_	*P*2_1_2_1_2	*P*2_1_2_1_2
Cell dimensions *a*, *b*, *c* (Å)	49.4, 56.4, 73.0	171.8, 171.8, 72.0	171.6, 171.6, 73.0	171.8, 171.8, 72.4	171.3, 171.3, 73.0	156.2, 156.2, 85.3	133.6, 141.8, 147.0	71.7, 162.5, 33.0	71.8, 163.0, 33.1
*α*, *β*, *γ* (°)	90, 104.6, 90	90, 90, 120	90, 90, 120	90, 90, 120	90, 90, 120	90, 90, 120	90, 90, 90	90, 90, 90	90, 90, 90
Resolution (Å)*	48–1.40 (1.50–1.40)	44–2.70 (2.80–2.70)	45–2.60 (2.70–2.60)	44–2.70 (2.80–2.70)	44–2.40 (2.50–2.40)	46–3.20 (3.30–3.20)	59–2.90 (3.00–2.90)	43–1.10 (1.20–1.10)	43–1.70 (1.80–1.70)
*R*_sym_	0.041 (0.815)	0.092 (1.743)	0.115 (1.789)	0.184 (2.210)	0.254 (3.589)	0.288 (2.696)	0.289 (3.680)	0.058 (0.221)	0.060 (0.078)
*I*/*σI*	18.1 (1.8)	16.3 (1.1)	12.3 (1.1)	12.3 (1.1)	12.3 (1.1)	7.7 (1.2)	13.6 (1.2)	23.9 (9.1)	24.0 (12.1)
Completeness (%)	99.1 (99.2)	99.9 (99.9)	99.9 (99.8)	99.9 (99.9)	99.7 (99.3)	99.9 (99.8)	99.9 (99.9)	96.2 (83.7)	91.7 (60.5)
Redundancy	3.7 (3.6)	7.2 (5.6)	6.5 (6.4)	8.6 (8.4)	19.6 (17.8)	12.0 (11.5)	14.6 (15.0)	11.9 (9.2)	6.3 (2.9)
*CC*(1/2)†	100.0 (68.7)	99.9 (38.0)	99.9 (45.3)	99.7 (44.6)	99.7 (58.4)	99.4 (36.3)	99.8 (48.4)	99.9 (97.6)	99.7 (98.6)
Resolution limit (Å) at *I/σI*=2	1.43	2.81	2.74	2.91	2.62	3.40	3.01	—	—
Wilson B-factor (Å^2^)	15.2	75.4	66.4	63.2	40.9	91.7	71.1	8.0	22.5
									
*Refinement*									
Resolution (Å)	1.40	2.70	—	—	2.40	3.20	2.90	1.10	—
No. reflections (all)	77,847	35,510	—	—	49,376	17,929	62,454	153,530	—
*R*_work/_ *R*_free_	0.18/0.23	0.18/0.23	—	—	0.20/0.24	0.24/0.29	0.19/0.24	0.13/0.15	—
Number of atoms									
Protein (all)	3,696	4,588	—	—	4,776	6,262	12,482	4,264	—
Ligand/ion	205	132	—	—	134	207	442	6	—
Water	350	0	—	—	182	0	0	838	—
B-factors									
Protein (all)	25.6	82.6	—	—	42.7	78.2	80.6	10.3	—
Ligand/ion	39.3	97.5	—	—	56.3	68.2	86.9	10.6	—
Water	37.0	—	—	—	43.7	—	—	22.7	—
R.m.s deviations									
Bond lengths (Å)	0.009	0.010	—	—	0.009	0.005	0.012	0.007	—
Bond angles (°)	2.48	1.39	—	—	1.37	1.25	1.78	1.25	—
Ramachandran favored (%)‡	97.1	97.6	—	—	97.4	95.5	95.2	96.4	—
Ramachandran outliers (%)	0	0	—	—	0	1	3	0	—
PDB accession code	4QI3	4QI4			4QI5	4QI6	4QI7	4QI8	

Only one crystal was used for each refined structure.

^*^Highest resolution shell is shown in parenthesis.

^†^Percentage of correlation between intensities from random half-datasets (Karplus, P. A., Diederichs, K.^47^). Values given represent correlations significant at the 0.1% level.

^‡^As determined by *MolProbity*^62^.

## References

[b1] Himmel M. E. . Biomass recalcitrance: engineering plants and enzymes for biofuel production. Science 315, 804–807 (2007). 17289988 10.1126/science.1137016

[b2] Gelfand I. . Sustainable bioenergy production from marginal lands in the US Midwest. Nature 493, 514–517 (2013). 23334409 10.1038/nature11811

[b3] Eriksson K.-E., Pettersson B. & Westermark U. Oxidation: an important enzyme reaction in fungal degradation of cellulose. FEBS Lett. 49, 282–285 (1974). 4474960 10.1016/0014-5793(74)80531-4

[b4] Bao W. J. & Renganathan R. Cellobiose oxidase of *Phanerochaete chrysosporium* enhances crystalline cellulose degradation by cellulases. FEBS Lett. 302, 77–80 (1992). 1587358 10.1016/0014-5793(92)80289-s

[b5] Henriksson G., Ander P., Pettersson B. & Pettersson G. Cellobiose dehydrogenase (cellobiose oxidase) from *Phanerochaete chrysosporium* as a wood degrading enzyme. Studies on cellulose, xylan and synthetic lignin. Appl. Microbiol. Biotechnol. 42, 790–796 (1995).

[b6] Dumonceaux T., Bartholomew K., Valeanu L., Charles T. & Archibald F. Cellobiose dehydrogenase is essential for wood invasion and nonessential for kraft pulp delignification by *Trametes versicolor*. Enzyme Microb. Technol. 29, 478–489 (2001).

[b7] Canam T., Town J. R., Tsang A., McAllister T. A. & Dumonceaux T. J. Biological pretreatment with a cellobiose dehydrogenase-deficient strain of *Trametes versicolor* enhances the biofuel potential of canola straw. Bioresource Technol. 102, 10020–10027 (2011). 10.1016/j.biortech.2011.08.045 21903381

[b8] Harris P. V. . Stimulation of lignocellulosic biomass hydrolysis by proteins of glycoside family 61: structure and function of a large enigmatic family. Biochemistry 49, 3305–3316 (2010). 20230050 10.1021/bi100009p

[b9] Vaaje-Kolstad G. . An oxidative enzyme boosting the enzymatic conversion of recalcitrant polysaccharides. Science 330, 219–222 (2010). 20929773 10.1126/science.1192231

[b10] Quinlan R. J. . Insights into the oxidative degradation of cellulose by a copper metalloenzyme that exploits biomass components. Proc. Natl Acad. Sci. USA 108, 15079–15084 (2011). 21876164 10.1073/pnas.1105776108 PMC3174640

[b11] Horn S. J., Vaaje-Kolstad G., Westereng B. & Eijsink V. G. Novel enzymes for the degradation of cellulose. Biotechnol. Biofuels 5, 45 (2011). 22747961 10.1186/1754-6834-5-45 PMC3492096

[b12] Agger J. W. . Discovery of LPMO activity on hemicelluloses shows the importance of oxidative processes in plant cell wall degradation. Proc. Natl Acad. Sci. USA 111, 6287–6292 (2014). 24733907 10.1073/pnas.1323629111 PMC4035949

[b13] Vu V. V., Beeson W. T., Span E. A., Farquhar E. R. & Marletta M. A. A family of starch-active polysaccharide monooxygenases. Proc. Natl Acad. Sci. USA 111, 13822–13827 (2014). 25201969 10.1073/pnas.1408090111 PMC4183312

[b14] Tian C. . Systems analysis of plant cell wall degradation by the model filamentous fungus *Neurospora crassa*. Proc. Natl Acad. Sci. USA 106, 22157–22162 (2009). 20018766 10.1073/pnas.0906810106 PMC2794032

[b15] Phillips C. M., Beeson W. T. IV, Cate J. H. & Marletta M. A. Cellobiose dehydrogenase and a copper-dependent polysaccharide monooxygenase potentiate cellulose degradation by *Neurospora crassa*. ACS Chem. Biol. 6, 1399–1406 (2011). 22004347 10.1021/cb200351y

[b16] Langston J. A. . Oxidoreductive cellulose depolymerization by the enzymes cellobiose dehydrogenase and glycoside hydrolase 61. Appl. Environ. Microbiol. 77, 7007–7015 (2011). 21821740 10.1128/AEM.05815-11 PMC3187118

[b17] Beeson W. T., Phillips C. M., Cate J. H. D. & Marletta M. A. Oxidative cleavage of cellulose by fungal copper-dependent polysaccharide monooxygenases. J. Am. Chem. Soc. 134, 890–892 (2011). 22188218 10.1021/ja210657t

[b18] Turbe-Doan A., Arfi Y., Record E., Estrada-Alvarado I. & Levasseur A. Heterologous production of cellobiose dehydrogenases from the basidiomycete *Coprinopsis cinerea* and the ascomycete *Podospora anserina* and their effect on saccharification of wheat straw. Appl. Microbiol. Biotechnol. 97, 4873–4885 (2013). 22940800 10.1007/s00253-012-4355-y

[b19] Payne C. M. . Fungal cellulases. Chem. Rev. 115, 1308–1448 (2015). 25629559 10.1021/cr500351c

[b20] Zamocky M. . Cellobiose dehydrogenase-a flavocytochrome from wood-degrading, phytopathogenic and saprotropic fungi. Curr. Protein Pept. Sci. 7, 255–280 (2006). 16787264 10.2174/138920306777452367

[b21] Harreither W. . Catalytic properties and classification of cellobiose dehydrogenases from ascomycetes. Appl. Environ. Microbiol. 77, 1804–1815 (2011). 21216904 10.1128/AEM.02052-10 PMC3067291

[b22] Henriksson G., Salumets A., Divne C. & Pettersson G. Studies of cellulose binding by cellobiose dehydrogenase and a comparison with cellobiohydrolase 1. Biochem. J. 324, 833–838 (1997). 9210407 10.1042/bj3240833 PMC1218499

[b23] Li X. . Structural basis for substrate targeting and catalysis by fungal polysaccharide monooxygenases. Structure 20, 1051–1061 (2012). 22578542 10.1016/j.str.2012.04.002 PMC3753108

[b24] Kim S., Ståhlberg J., Sandgren M., Paton R. S. & Beckham G. T. Quantum mechanical calculations suggest that lytic polysaccharide monooxygenases use a copper-oxyl, oxygen-rebound mechanism. Proc. Natl Acad. Sci. USA 111, 149–154 (2014). 24344312 10.1073/pnas.1316609111 PMC3890868

[b25] Hallberg B. M. . A new scaffold for binding haem in the cytochrome domain of the extracellular flavocytochrome cellobiose dehydrogenase. Structure 8, 79–88 (2000). 10673428 10.1016/s0969-2126(00)00082-4

[b26] Hallberg B. M., Henriksson G., Pettersson G. & Divne C. Crystal structure of the flavoprotein domain of the extracellular flavocytochrome cellobiose dehydrogenase. J. Mol. Biol. 315, 421–434 (2002). 11786022 10.1006/jmbi.2001.5246

[b27] Page C. C., Moser C. C., Chen X. & Dutton P. L. Natural engineering principles of electron tunnelling in biological oxidation–reduction. Nature 402, 47–52 (1999). 10573417 10.1038/46972

[b28] Lederer F. Another look at the interaction between mitochondrial cytochrome *c* and flavocytochrome *b*2. Eur. Biophys. J. 40, 1283–1299 (2011). 21503671 10.1007/s00249-011-0697-0

[b29] Hallberg B. M., Henriksson G., Pettersson G., Vasella A. & Divne C. Mechanism of the reductive half-reaction in cellobiose dehydrogenase. J. Biol. Chem. 278, 7160–7166 (2003). 12493734 10.1074/jbc.M210961200

[b30] Bernado P., Mylonas E., Petoukhov M. V., Blackledge M. & Svergun D. I. Structural Characterization of flexible proteins using small-angle X-ray scattering. J. Am. Chem. Soc. 129, 5656–5664 (2007). 17411046 10.1021/ja069124n

[b31] Petoukhov M. V. . New developments in the ATSAS program package for small-angle scattering data analysis. J. Appl. Crystallogr. 45, 342–350 (2012). 25484842 10.1107/S0021889812007662 PMC4233345

[b32] Eibinger M. . Cellulose surface degradation by a lytic polysaccharide monooxygenase and its effect on cellulose hydrolytic efficiency. J. Biol. Chem. 289, 35929–35938 (2014). 25361767 10.1074/jbc.M114.602227 PMC4276861

[b33] Wu M. . Crystal structure and computational characterization of the lytic polysaccharide monooxygenase GH61D from the basidiomycota fungus *Phanerochaete chrysosporium*. J. Biol. Chem. 288, 12828–12839 (2013). 23525113 10.1074/jbc.M113.459396 PMC3642327

[b34] Forsberg Z. . Structural and functional characterization of a conserved pair of bacterial cellulose-oxidizing lytic polysaccharide monooxygenases. Proc. Natl Acad. Sci. USA 111, 8446–8451 (2014). 24912171 10.1073/pnas.1402771111 PMC4060697

[b35] Guallar V. Haem electron transfer in peroxidases: the propionate e-pathway. J. Phys. Chem. B 112, 13460–13464 (2008). 10.1021/jp806435d 18816089

[b36] Zamocky M. . Cloning, sequence analysis and heterologous expression in *Pichia pastoris* of a gene encoding a thermostable cellobiose dehydrogenase from *Myriococcum thermophilum*. Protein Express. Purif. 59, 258–265 (2008). 10.1016/j.pep.2008.02.007 18374601

[b37] Levasseur A., Drula E., Lombard V., Coutinho P. M. & Henrissat B. Expansion of the enzymatic repertoire of the CAZy database to integrate auxiliary redox enzymes. Biotechnol. Biofuels 6, 41 (2013). 23514094 10.1186/1754-6834-6-41 PMC3620520

[b38] Kittl R., Kracher D., Burgstaller D., Haltrich D. & Ludwig R. Production of four *Neurospora crassa* lytic polysaccharide monooxygenases in *Pichia pastoris* monitored by a fluorimetric assay. Biotechnol. Biofuels 5, 79 (2012). 23102010 10.1186/1754-6834-5-79 PMC3500269

[b39] Sygmund C. . Characterization of the two *Neurospora crassa* cellobiose dehydrogenases and their connection to oxidative cellulose degradation. Appl. Environ. Microbiol. 78, 6161–6171 (2012). 22729546 10.1128/AEM.01503-12 PMC3416632

[b40] Flitsch A. . Cellulose oxidation and bleaching processes based on recombinant *Myriococcum thermophilum* cellobiose dehydrogenase. Enzyme Microb. Technol. 52, 60–67 (2013). 23199740 10.1016/j.enzmictec.2012.10.007

[b41] Kabsch W. Automatic processing of rotation diffraction data from crystals of initially unknown symmetry and cell constants. J. Appl. Crystallogr. 26, 795–800 (1993).

[b42] McCoy A. J. . Phaser crystallographic software. J. Appl. Crystallogr. 40, 658–674 (2007). 19461840 10.1107/S0021889807021206 PMC2483472

[b43] Adams P. D. . PHENIX: a comprehensive Python-based system for macromolecular structure solution. Acta Crystallogr. D 66, 213–221 (2010). 20124702 10.1107/S0907444909052925 PMC2815670

[b44] Vonrhein C., Blanc E., Roversi P. & Bricogne G. Automated structure solution with autoSHARP. Methods Mol. Biol. 364, 215–230 (2007). 17172768 10.1385/1-59745-266-1:215

[b45] Jones T. A., Zou J. Y., Cowan S. W. & Kjeldgaard M. Improved methods for building protein models in electron density maps and the location of errors in these models. Acta Crystallogr. A 47, 110–119 (1991). 2025413 10.1107/s0108767390010224

[b46] Emsley P. & Cowtan K. Coot: model-building tools for molecular graphics. Acta Crystallogr. D 60, 2126–2132 (2004). 15572765 10.1107/S0907444904019158

[b47] Karplus P. A. & Diederichs K. Linking crystallographic model and data quality. Science 336, 1030–1033 (2012). 22628654 10.1126/science.1218231 PMC3457925

[b48] Long F., Vagin A. A., Young P. & Murshudov G. N. BALBES: a molecular replacement pipeline. Acta Crystallogr. D 64, 125–132 (2008). 18094476 10.1107/S0907444907050172 PMC2394813

[b49] Langer G., Cohen S. X., Lamzin V. S. & Perrakis A. Automated macromolecular model building for X-ray crystallography using ARP/wARP version 7. Nat. Protoc. 3, 1171–1179 (2008). 18600222 10.1038/nprot.2008.91 PMC2582149

[b62] Sheldrick G. M. Experimental phasing with SHELXC/D/E: combining chain tracing with density modification. Acta Crystallogr. D 66, 479–485 (2010). 20383001 10.1107/S0907444909038360 PMC2852312

[b51] Collaborative Computational Project, Number 4. The CCP4 suite: programs for protein crystallography. Acta Crystallogr. D 50, 760–763 (1994). 15299374 10.1107/S0907444994003112

[b52] Murshudov G. N. . REFMAC5 for the refinement of macromolecular crystal structures. Acta Crystallogr. D 67, 355–367 (2011). 21460454 10.1107/S0907444911001314 PMC3069751

[b50] Terwilliger T. C. Automated main-chain model building by template matching and iterative fragment extension. Acta Crystallogr. D 59, 38–44 (2003). 12499537 10.1107/S0907444902018036 PMC2745878

[b53] Hura G. L. . Robust, high-throughput solution structural analyses by small angle X-ray scattering (SAXS). Nat. Method 6, 606–612 (2009). 10.1038/nmeth.1353 PMC3094553 19620974

[b54] Putnam C. D., Hammel M., Hura G. L. & Tainer J. A. X-ray solution scattering (SAXS) combined with crystallography and computation: Defining accurate macromolecular structures, conformations and assemblies in solution. Q. Rev. Biophys. 40, 191–285 (2007). 18078545 10.1017/S0033583507004635

[b55] Labrador A., Cerenius Y., Svensson C., Keld T. & Plivelic T. The yellow mini-hutch for SAXS experiments at MAX IV Laboratory. J. Phys.: Conf. Series 425, 072019 (2013).

[b56] Konarev P. V., Volkov V. V., Sokolova A. V., Koch M. H. J. & Svergun D. I. PRIMUS: A Windows PC-based system for small-angle scattering data analysis. J. Appl. Crystallogr. 36, 1277–1282 (2003).

[b57] Tria G., Mertens H. D. T., Kachala M. & Svergun D. I. Advanced ensemble modelling of flexible macromolecules using X-ray solution scattering. IUCrJ 2, 207–217 (2015). 10.1107/S205225251500202X PMC4392415 25866658

[b58] de Vries S. J. . HADDOCK versus HADDOCK: New features and performance of HADDOCK2.0 on the CAPRI targets. Proteins: Struc. Funct. Bioinformatics 69, 726–733 (2007). 10.1002/prot.21723 17803234

[b59] de Vries S. J., van Dijk M. & Bonvin A. M. J. J. The HADDOCK web server for data-driven biomolecular docking. Nat. Protoc. 5, 883–897 (2010). 20431534 10.1038/nprot.2010.32

[b60] Wassenaar, . WeNMR: structural biology on the grid. J. Grid. Comp. 10, 743–767 (2012).

[b61] Vincent B. . MolProbity: all-atom structure validation for macromolecular crystallography. Acta Crystallogr. D. 66, 12–21 (2010). 20057044 10.1107/S0907444909042073 PMC2803126

