# Supplementary material for: Structural basis for cellobiose dehydrogenase action during oxidative cellulose degradation
Source: Nat Commun. 2015 Jul 7;6:7542. doi: 10.1038/ncomms8542 (PMC4507011; doi:10.1038/ncomms8542)
Supplement: Supplementary Information — Supplementary Figures 1-11 [file ncomms8542-s1.pdf]

Supplementary Information

```
MtCDH QNNVPNTFTDPDSGITFNTWGLDEDSPQTQGGFTFGVALPSDAL-TTDASEFIGYLKCARN  60
NcCDH  QTAPKTFTHPDTGIVFNTWSASD--SQTKGGFTVGMALPSNAL-TTDATEFIGYLECSSA  57
PcCDH   QSASQFTDPTTGFQFTGITDPV------HDVTYGFVFPPLATSGAQSTEFIGEVVAPIA  53

MtCDH D---ESGWCGISLGGPMTNSLLITAWPHEDTVYTSLRFATGYAMPDVYEGDAEITQV-SSS 117
NcCDH KNGANSGWCGVSLRGAMTNNLLITAWPSDGEVYTNLMFATGYAMPKNYAGDAKITQI-ASS 117
PcCDH -----SKWIGIALGGAMNNDLLLVAWANGNQIVSSTRWATGYVQPTAYTGTATLTTLPETT 109

MtCDH VNSTHFSLIFRCKNCLQWSHGGSSGGASTSGGVLVLGWVQAFDDPGNPTCPEQITLQQHDN 178
NcCDH VNATHFTLVFRCQNCLSWDQDGVTGGISTSNKGAQLGWVQAFPSPGNPTCPTQITLSQHDN 178
PcCDH INSTHWKWVFRCQGCTEWN---NGGGIDVTS-QGVLAWAFSNVAVDDPSDP-QSTFSEHT- 164

MtCDH GMGIWGAQLNTDAASPSYTDWAAQATKTVTGDCEGPTETSV--VGVPVPTGVSFDYIVVGG 237
NcCDH GMGQWGAAFDSNIANPSYTAWAAKATKTVTGTCSGPVTTSI--AATPVPTGVSFDYIVVGG 237
PcCDH DFGFFGID-YSTAHSANYQNYLNGDSGNPTTTSTKPTSTSSSVTTGPTVSATPYDYIIVGA 224

MtCDH GAGGIPAADKLSEAGKSVLLIEKGFASTANTGGTLGPEWLEGHDLTRFDVPGLCNQIWVD- 297
NcCDH GAGGIPVADKLSESGKSVLLIEKGFASTGEHGGTLKPEWLNNTSLTRFDVPGLCNQIWKD- 297
PcCDH GPGGIIAADRLSEAGKKVLLLERGGPSTKQTGGTYVAPWATSSGLTKFDIPGLFESLFTDS 285

MtCDH SKGIACEDTDQMAGCVLGGGTAVNAGLWFKPYSLDWDYLFPDGWKYNDVQPAINRALSRIP 358
NcCDH SDGIACSDTDQMAGCVLGGGTAINAGLWYKPYTKDWDYLFPSGWKGSDIAGATSRALSRIP 358
PcCDH NPFWWCKDITVFAGCLVGGGTSVNGALYWYPNDGDFSSSVGWPSSWTNHAPYTSKLSSRLP 346

MtCDH GTDAPSTDGKRYYQEGFEVLSKGLAAGGWTSVTANNAPDKKNRTFAHAPFMFAGGERNGPL 419
NcCDH GTTTPSQDGKRYLQQGFEVLANGLKASGWKEVDSLKDSEQKNRTFSHTSYMYINGERGGPL 419
PcCDH STDHPSTDGQRYLEQSFNVVSQLLKGQGYNQATINDNPNYKDHVFGYSAFDFLNGKRAGPV 407

MtCDH GTYFQTAKKRNNFDVWLNTSVKRVIREGGHITGVEVEPFRDGGYEGIVPVTKVTGRVILSA 480
NcCDH ATYLVSAKKRSNFKLWLNTAVKRVIREGGHITGVEVEAFRNGGYSGIIPVTNTTGRVVLSA 480
PcCDH ATYLQTALARPNFTFKTNVMVSNVVRNGSQILGVQTNDPTLG-PNGFIPVTP-KGRVILSA 466

MtCDH GTFGSAKILLRSGIGPEDQLEVVAASEKDGPTMIGNSSWINLPVGYNLDDHLNTDTVISHP 541
NcCDH GTFGSAKILLRSGIGPKDQLEVVKAS-ADGPTMVSNSSWIDLPVGHNLVDHTNTDTVIQHN 540
PcCDH GAFGTSRILFQSGIGPTDMIQTVQSNPTAAAALPPQNQWINLPVGMNAQDNPSINLVFTHP 527

MtCDH DVVFYD-FYEAWDDPIESDKNSYLESRTGILAQAAPNIGPMFWEEIVGADGIVRQLQWTAR 601
NcCDH NVTFYD-FYKAWDNPNTTDMNLYLNGRSGIFAQAAPNIGPLFWEEITGADGIVRQLHWTAR 600
PcCDH SIDAYENWADVWSNPRPADAAQYLANQSGVFAGA--SPKLNFWRAYSGSDGFTRYAQGTVR 586

MtCDH VEGSLGAP-----NGHTMTMSQYLGRGATSRGRMTITPSLTTIVSDVPYLKDPNDKEAVIQ 657
NcCDH VEGSFETP-----DGYAMTMSQYLGRGATSRGRMTLSPTLNTVVSDLPYLKDPNDKAAVVQ 656
PcCDH PGAASVNSSLPYNASQIFTITVYLSTGIQSRGRIGIDAALRGTVLTPPWLVNPVDKTVLLQ 647

MtCDH GIINLQNALQNVANLTWLFPNSTITPREYVESMVVSPSNRRSNHWMGTNKLGTDDGRKGGS 718
NcCDH GIVNLQKALANVKGLTWAYPSANQTAADFVDKQPVTYQSRRSNHWMGTNKMGTDDGRSGGT 717
PcCDH ALHDVVSNIGSIPGLTMITPDVTQTLEEYVDAYDP--ATMNSNHWVSSTTIGSSP----QS 702

MtCDH AVVDLDTRVYGTDNLFVIDASIFPGVPTTNPTSYIVVAAEHASSRILALPDLEPVPKYGQC 779
NcCDH AVVDTNTRVYGTDNLYVVDASIFPGVPTTNPTAYIVVAAEHAAAKILAQPANEAVPKWGWC 778
PcCDH AVVDSNVKVFGTNNLFIVDAGIIPHLPTGNPQGTLMSAAEQAAAKILALAGGP-------- 755

MtCDH GGREWTGSFVCADGSTCEYQNEWYSQCL 807
NcCDH GGPTYTGSQTCQAPYKCEKQNDWYWQCV 806
PcCDH ----------------------------
```

Supplementary Figure 1 | Structure-based sequence alignment of CDHs with known structures. Amino-acid sequence alignment based on a least-squares superpositioning of the structures for ascomycete *Mt*CDH (UniProt A9XK88) and *Nc*CDH (UniProt Q7RXM0), and the basidiomycete *Pc*CDH (PDB 1KDG; UniProt Q01738). Amino-acid identities across two and three sequences are colored yellow and white on gray background, respectively. The colored bars above the alignment outline the domain boundaries: cytochrome domain, red; linker, green; dehydrogenase domain, blue; and cellulose-binding domain, orange. The asterisks denote important residues discussed in the text: the principal heme-binding amino acids in the cytochrome domain (red; *Mt*CDH Met74, Tyr99, His176); catalytic amino acids in the flavodehydrogenase domain (green; His701, Asn748); amino acids that interact with the sugar electron-donor substrate in the dehydrogenase (blue; Trp295, Arg601, Glu603, Tyr619, Asn700); and cellulose-binding amino acids (orange; Glu771, Lys775, Tyr776, Gln778, Glu783, Gln799, Asn800, Trp802, Tyr803, Gln805) present in the type-1 CBM of *Mt*CDH and *Nc*CDH (*Pc*CDH binds to cellulose but lacks a CBM); and additional residues targeted for IET analysis (gray; Asn292, Ser298, Met309, Arg698). The domain organization in the ascomycete CDHs are as follows (*Mt*CDH numbering): cytochrome domain, residues 1–206; linker, residues 207–219; flavodehydrogenase domain, 220–771; CBM, 772–807. Pairwise structural identities for the amino-acid sequences are: *Mt*CDH–*Nc*CDH, 69%; *Mt*CDH-*Pc*CDH, 36%; *Nc*CDH–*Pc*CDH, 35%. The N-terminal signal-peptide sequences are not included.

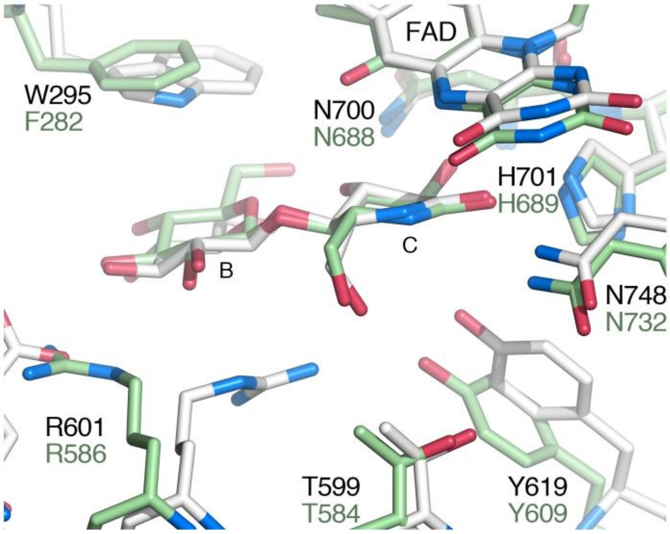

Supplementary Figure 2 | Binding of CBLM to *Mt*CDH and *Pc*CDH. Overlay of the active sites in the CBLM-bound DH domains of *Mt*CDH (white) and *Pc*CDH (green). Binding of CBLM to DH in *Pc*CDH (PDB code 1NAA) is nearly identical to that in *Mt*CDH, but with the B-site Trp295 in *Mt*CDH replaced by a phenylalanine. The reaction is initiated by abstraction of the substrate O1 hydroxyl proton by a catalytic histidine (His689) concomitantly with FAD reduction by the transfer of the C1 hydrogen as hydride to the flavin N5 atom.

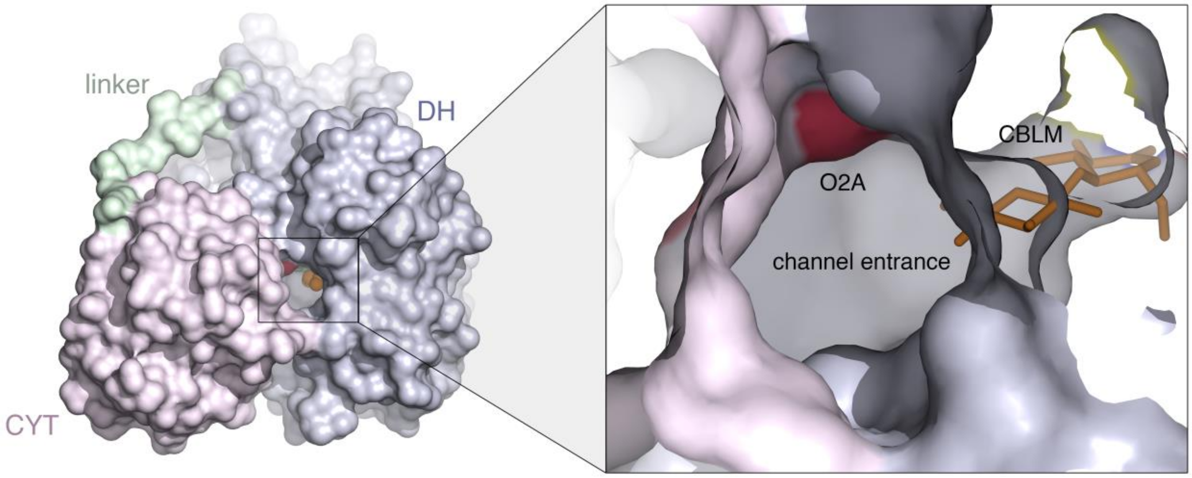

Supplementary Figure 3 | Surface channel between CYT and DH in *Mt*CDH. Surface representation of the closed *Mt*CDH state showing the channel formed between the CYT and DH domains (left). The view is from the top with the CBM not visible. The channel leads to the active-site entrance in DH (right). The O2A atom of heme *b* propionate-A and the docked CBLM molecule are shown in red and orange, respectively. *N*-linked glycans are not shown.

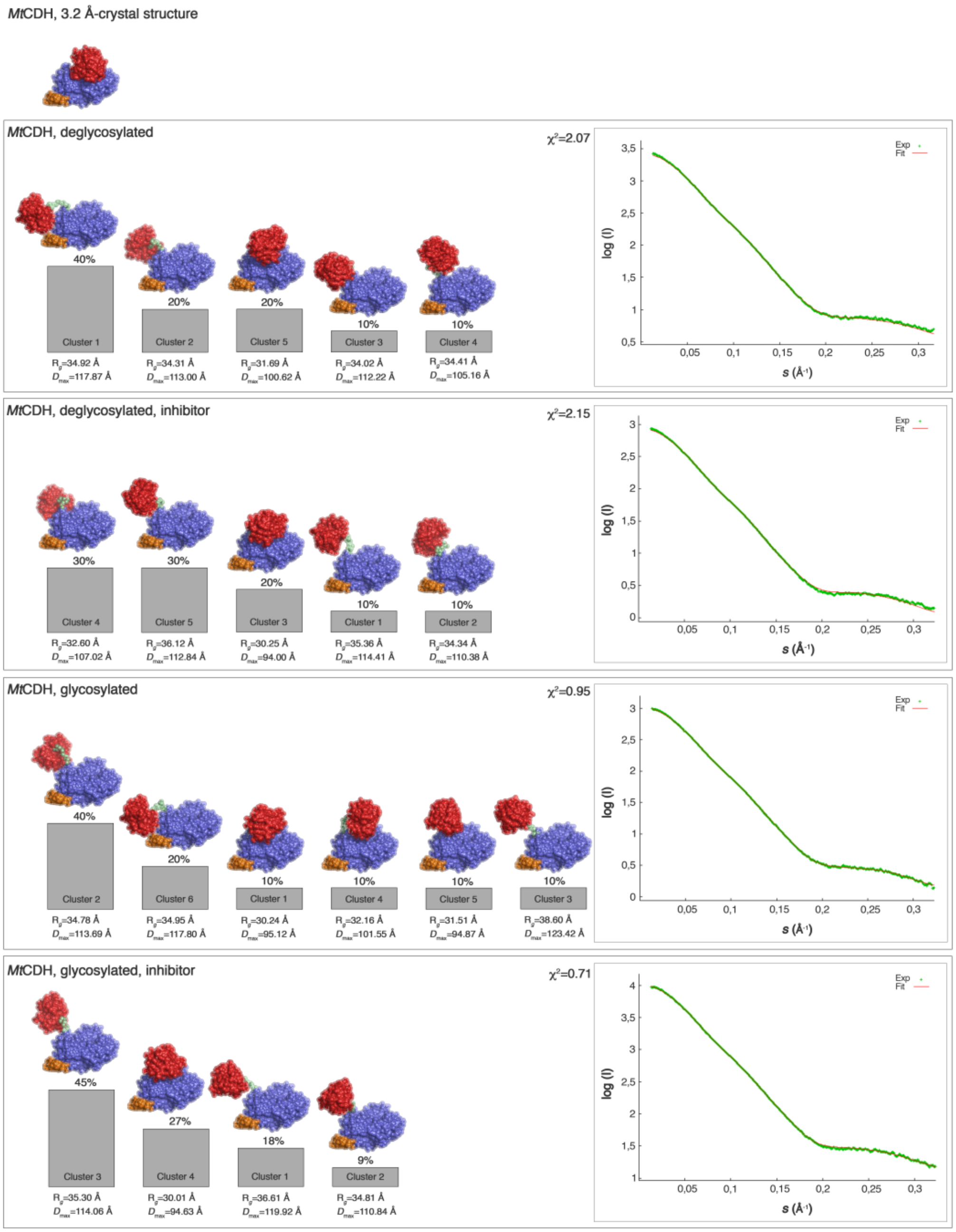

Supplementary Figure 4 | SAXS modeling for *Mt*CDH. Left: Conformational clusters obtained using EOM analysis for deglycosylated and glycosylated *Mt*CDH in the absence or presence of the inhibitor CBLM. The approximate fraction of each conformer in the final ensemble is given as a percentage. Color coding: CYT domain (red), DH domain (blue), linker (green), CBM (orange). Right: Experimental SAXS scattering curves (green line), and the fit of the EOM ensembles represented by the conformational clusters to the scattering curves (red line); $s=4\pi \sin \theta/\lambda$.

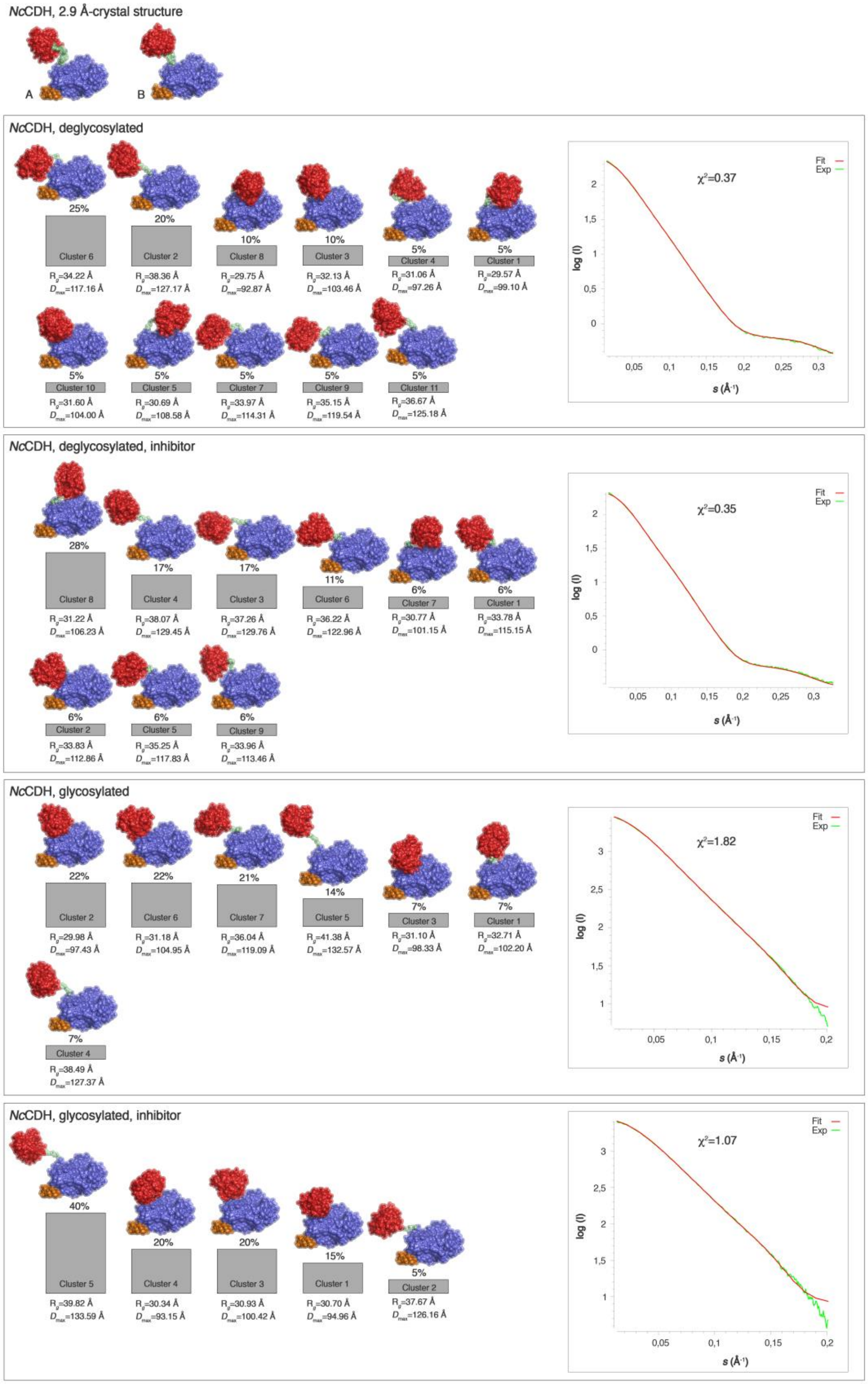

Supplementary Figure 5 | SAXS modeling for *Nc*CDH. Information as for *Mt*CDH in Supplementary Figure 4.

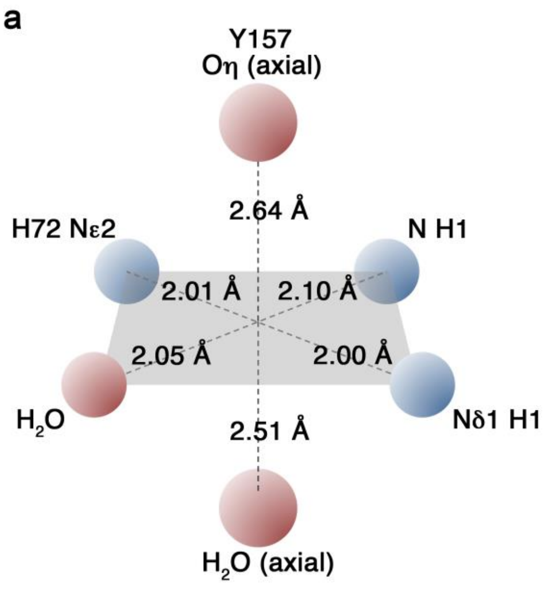

Supplementary Figure 6 | Coordination geometry in *Nc*LPMO$_{9F}$. The coordination shell of the six-coordinated copper is distorted octahedral with *mer*-[MA_3B_3] geometry in both *Nc*LPMO$_{9F}$ molecules of the asymmetric unit. Nitrogen ligands are shown as blue and oxygen ligands as red spheres, respectively. Equatorial metal ligands reside in the shaded plane with the axial ligands perpendicular to the plane. The axial coordination distances are elongated indicating Jahn-Teller distortion, but slightly longer than observed in inorganic complexes. (**a**) *Nc*LPMO$_{9F}$ molecule A: two water molecules donate oxygen atoms for metal coordination where one serves as the second axial ligand, and the other as the fourth equatorial ligand. (**b**) *Nc*LPMO$_{9F}$ molecule B: the two water-oxygen ligands in (a) have been replaced by two carboxylate oxygens donated by Asp33.

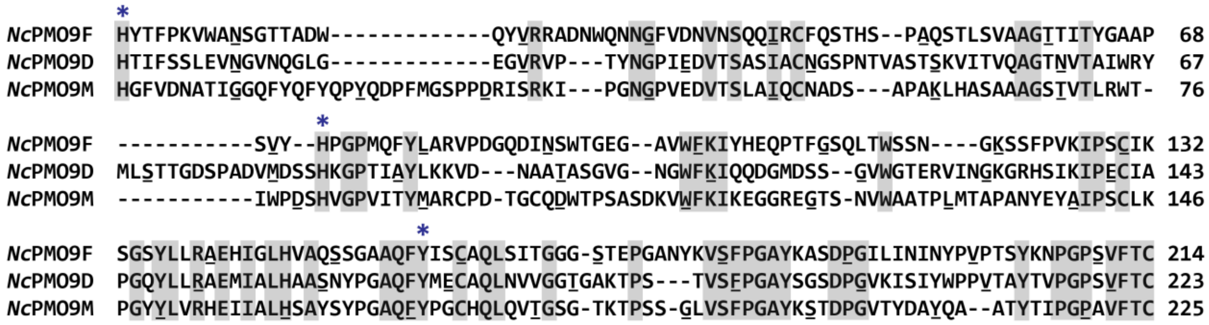

Supplementary Figure 7 | Sequence alignment of three *N. crassa* LPMOs. Amino-acid sequence alignment for *Nc*LPMO$_{9F}$, *Nc*LPMO$_{9D}$ and *Nc*LPMO$_{9M}$. Conserved residues are shaded, and every tenth residue is underlined. The copper-coordinating residues are marked with blue asterisks.

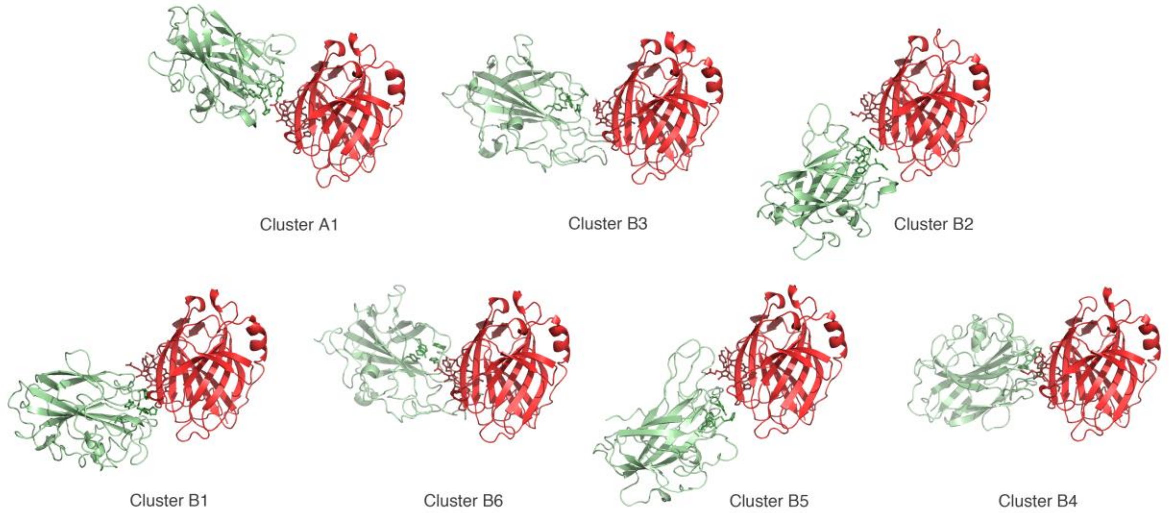

Surface	Cluster	N	H-score	r.m.s.d.	E_{vdw} (kcal mol^{-1})	E_{elec} (kcal mol^{-1})	E_{desolv} (kcal mol^{-1})	E_{restr_viol} (kcal mol^{-1})	BSA (Å^2)	Z-score
A	1	197	–183.7 ± 1.0	1.2 ± 0.8	32.2 ± 4.9	–1039.5 ± 19.9	–7.9 ± 7.3	0.0 ± 0.00	704.6 ± 27.9	0
B	3	21	–177.6 ± 8.3	4.2 ± 2.4	24.8 ± 3.8	–1018.2 ± 34.4	1.2 ± 11.1	0.7 ± 0.55	835.1 ± 49.8	–1.7
B	2	60	–125.5 ± 4.7	10.6 ± 1.0	–25.8 ± 4.7	–472.0 ± 26.4	–5.3 ± 12.0	0.2 ± 0.32	1304.3 ± 61.8	–0.2
B	1	96	–120.4 ± 7.4	7.5 ± 0.3	–29.1 ± 11.5	–452.8 ± 63.8	–0.7 ± 2.9	0.0 ± 0.02	1374.0 ± 103.1	–0.1
B	6	4	–114.8 ± 5.5	2.2 ± 0.4	–0.3 ± 2.5	–578.3 ± 17.2	1.1 ± 2.6	0.3 ± 0.33	821.8 ± 40.3	0.1
B	5	4	–109.6 ± 13.3	12.0 ± 0.8	–12.5 ± 4.9	–487.4 ± 61.6	0.3 ± 4.9	0.3 ± 0.59	997.7 ± 153.0	0.2
B	4	6	–55.9 ± 1.7	13.6 ± 0.3	–27.0 ± 3.3	–132.1 ± 25.0	–2.5 ± 5.4	0.0 ± 0.00	955.6 ± 85.9	1.7

Supplementary Figure 8 | CYT-LPMO models generated by *HADDOCK* docking. Automatically generated models of *Nc*CYT and *Nc*LPMO$_{9F}$ by *HADDOCK*. Only CYT and LPMO are shown. The heme group in CYT and the copper-binding residues in *Nc*LPMO$_{9F}$ are shown as stick objects. All *HADDOCK* clusters orient the CYT heme group towards the copper-binding edge of LPMO but with different relative rotation.

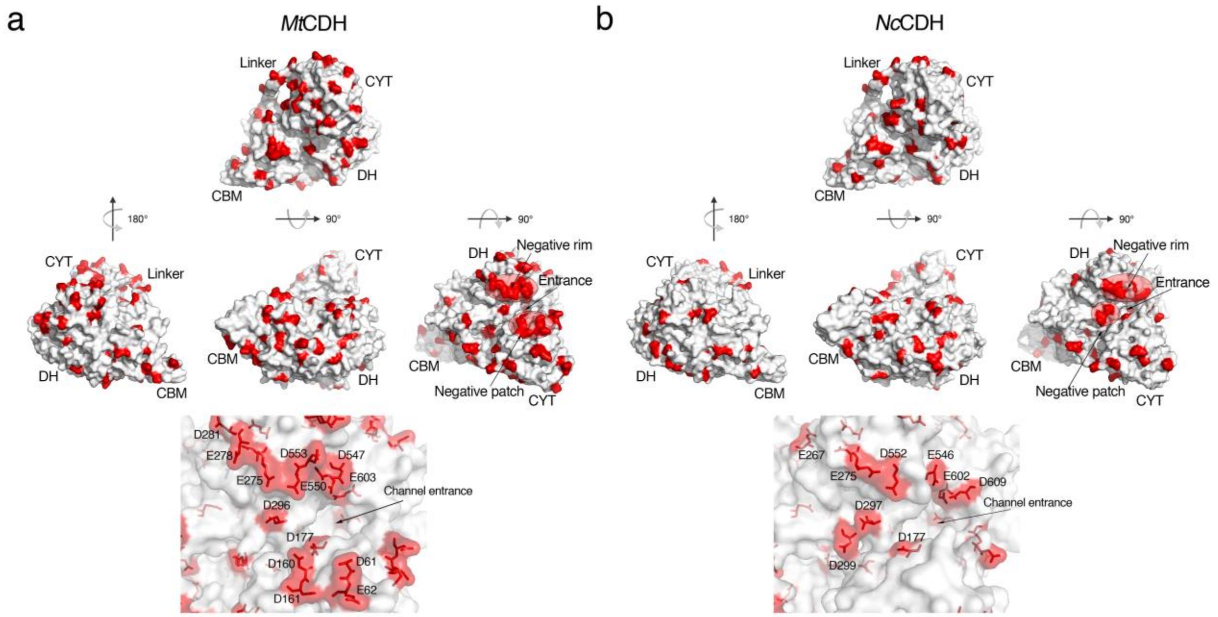

Supplementary Figure 9 | Negatively charged residues at the *Mt*CDH and *Nc*CDH surfaces. (**a**) Surface distribution of aspartate and glutamate residues (red) for *Mt*CDH in the closed state as observed in the crystal structure. *Top*, view showing one side of *Mt*CDH with evenly distributed negative charges; *left*, the opposite side with evenly placed Asp and Glu; *middle*, the bottom face that would be oriented towards a cellulose surface shows no clustering of negative charges; *right*, view from the top where the channel entrance between CYT and DH is located. Negatively charged regions flank the entrance on both sides creating an Asp/Glu rim on the DH side, and a patch with Asp and Glu on the CYT side. *Bottom panel*, zoom in on the negative rim on DH including side chains (from left to right): Asp286, Glu414 (not visible in the picture), Asp281, Glu278, Glu275, Asp553, Glu550, Asp547 and Glu603 in the DH domain. The patch on CYT includes Asp61, Glu62, Asp160, Asp161, and Asp177. (**b)** Distribution of aspartate and glutamate residues (red) on the surface of the modeled closed state of *Nc*CDH. The four views are identical to those in panel (a) above. As for *Mt*CDH, an Asp/Glu rim is located on the DH side and an Asp/Glu patch on the CYT side. Residues defining the negative Asp/Glu rim on DH (from left to right): Glu267, Glu275, Asp552, Asp546, Glu602 and Asp609. Residues defining the negative Asp/Glu patch on CYT: Asp297, Asp299 and Asp177.

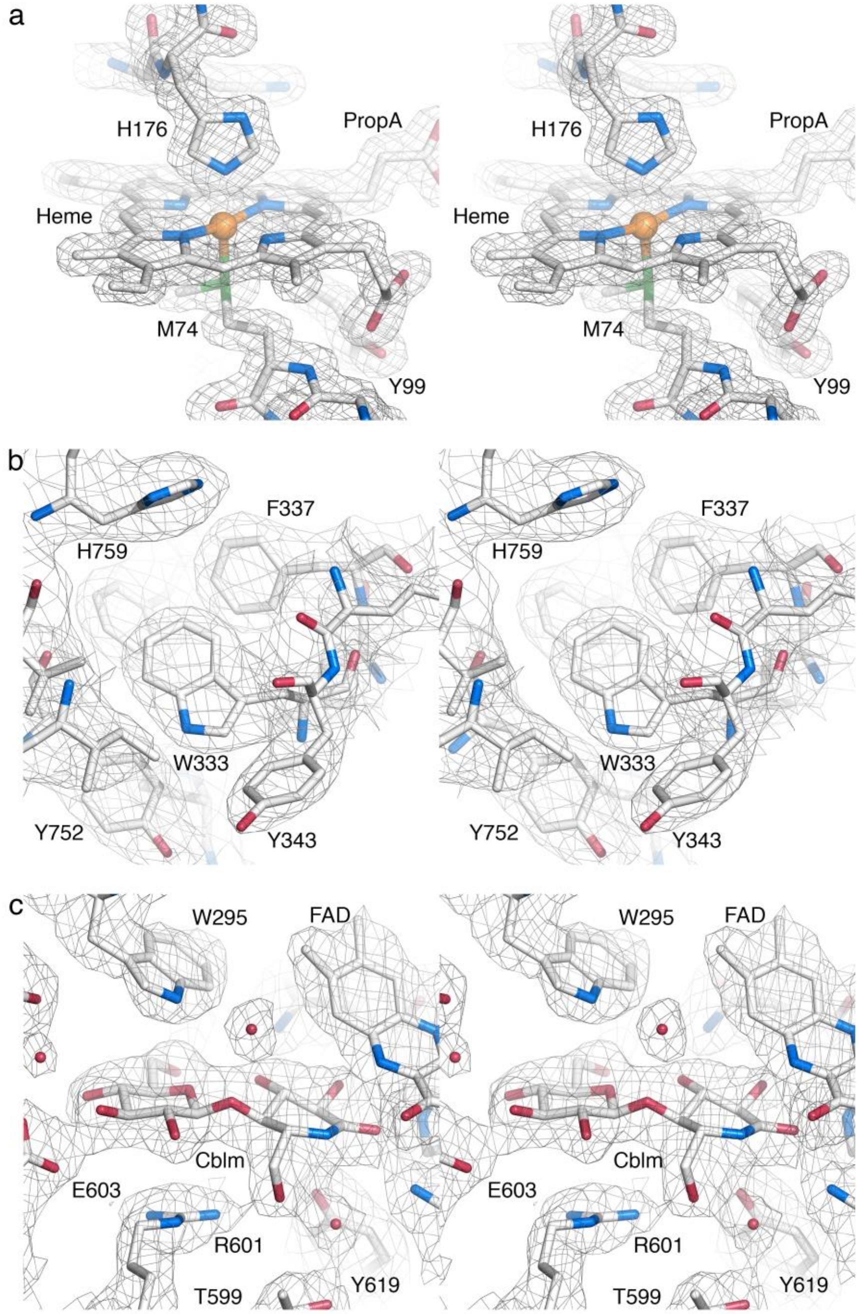

Supplementary Figure 10 | Quality of the electron density for *Mt*CYT, *Mt*DH and *Mt*DH-CBLM. Stereo images showing $2F_o$-F_c electron density for (**a**) *Mt*CYT calculated at 1.40 Å resolution and contoured at 1.2σ; (**b**) *Mt*DH calculated at 2.70 Å resolution and contoured at 1.7σ; *Mt*DH-CBLM complex calculated at 2.40 Å resolution and contoured at 1.5σ.

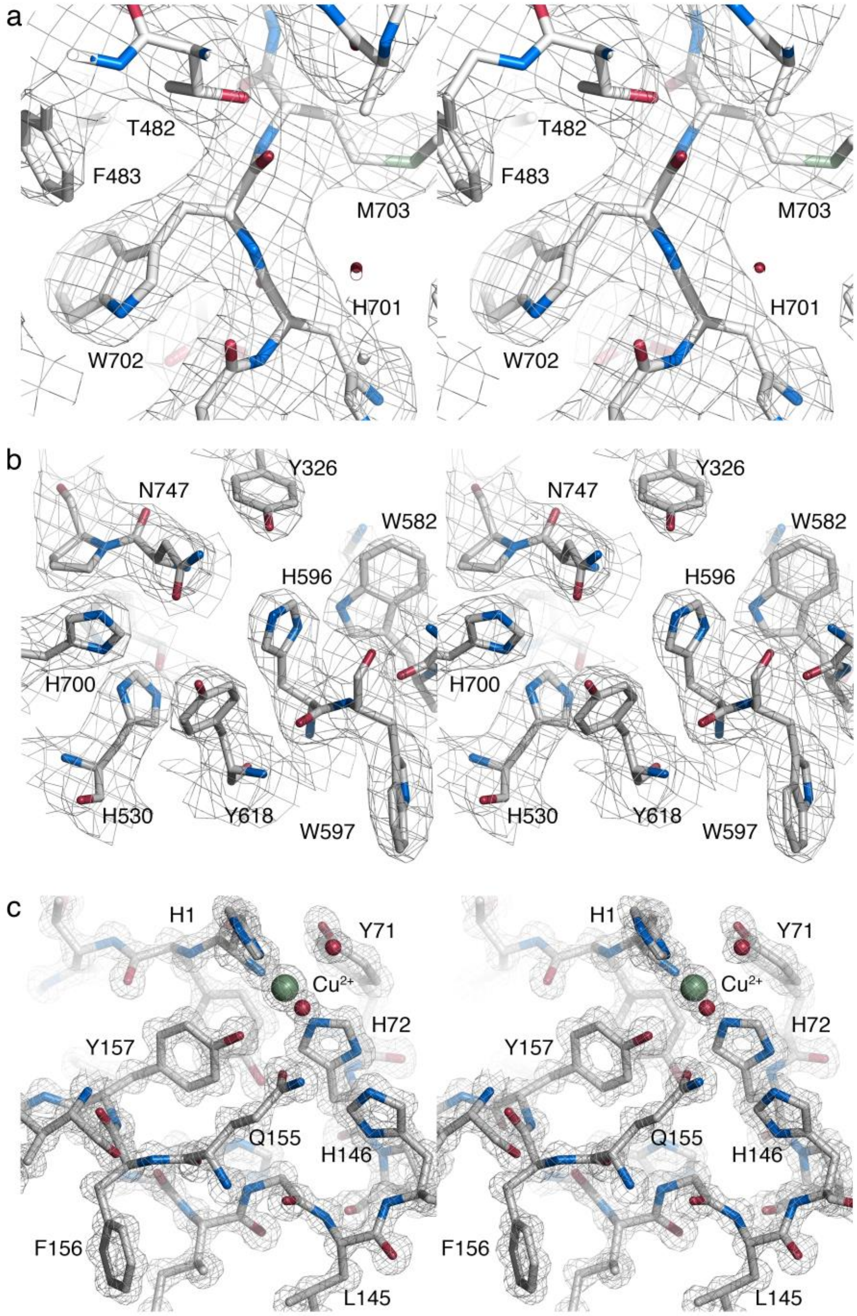

Supplementary Figure 11 | Quality of the electron density for *Mt*CDH, *Nc*CDH and *Nc*LPMO$_{9F}$. Stereo images showing 2F_o-F_c electron density for (**a**) *Mt*CDH calculated at 3.20 Å resolution and contoured at 0.8σ; (**b**) *Nc*CDH calculated at 2.90 Å resolution and contoured at 1.0σ; *Nc*LPMO$_{9F}$ calculated at 1.10 Å resolution and contoured at 1.7σ.
